# Evolution of Linoleic Acid Biosynthesis Paved the Way for Ecological Success of Termites

**DOI:** 10.1093/molbev/msad087

**Published:** 2023-04-12

**Authors:** Stanislav Macháček, Michal Tupec, Natan Horáček, Martina Halmová, Amit Roy, Aleš Machara, Pavlína Kyjaková, Ondřej Lukšan, Iva Pichová, Robert Hanus

**Affiliations:** Viral and Microbial Proteins, Institute of Organic Chemistry and Biochemistry of the Czech Academy of Sciences, Prague, Czech Republic; Department of Biochemistry and Microbiology, University of Chemistry and Technology Prague, Prague, Czech Republic; Viral and Microbial Proteins, Institute of Organic Chemistry and Biochemistry of the Czech Academy of Sciences, Prague, Czech Republic; Faculty of Science, Charles University, Prague, Czech Republic; Faculty of Science, Charles University, Prague, Czech Republic; Chemistry of Social Insects, Institute of Organic Chemistry and Biochemistry of the Czech Academy of Sciences, Prague, Czech Republic; Viral and Microbial Proteins, Institute of Organic Chemistry and Biochemistry of the Czech Academy of Sciences, Prague, Czech Republic; Faculty of Science, Charles University, Prague, Czech Republic; Forest Molecular Entomology Lab, Czech University of Life Sciences, Prague, Czech Republic; Drug Discovery, Institute of Organic Chemistry and Biochemistry of the Czech Academy of Sciences, Prague, Czech Republic; Chemistry of Social Insects, Institute of Organic Chemistry and Biochemistry of the Czech Academy of Sciences, Prague, Czech Republic; Chemistry of Social Insects, Institute of Organic Chemistry and Biochemistry of the Czech Academy of Sciences, Prague, Czech Republic; Viral and Microbial Proteins, Institute of Organic Chemistry and Biochemistry of the Czech Academy of Sciences, Prague, Czech Republic; Chemistry of Social Insects, Institute of Organic Chemistry and Biochemistry of the Czech Academy of Sciences, Prague, Czech Republic

**Keywords:** fatty acyl desaturases, linoleic acid, biosynthesis, termites, Isoptera, Blattodea

## Abstract

Termites are dominant animals of tropical terrestrial ecosystems. Their success is due to their eusocial organization as well as their ability to digest dead plant tissues. While being extremely abundant, the termite diet is poor in crucial nutrients, such as fatty acids. Linoleic acid (LA) is a precursor for many vital biomolecules, and most animals depend on its dietary supply. Termites count among the exceptions known to produce LA de novo, presumably via the action of an unknown Δ12 fatty acyl desaturase (FAD) introducing the second double bond into monounsaturated oleic acid. Here, we search for the evolutionary origin of LA biosynthesis in termites. To this end, we compile the repertoire of FAD homologs from 57 species of termites and their closest relatives, the cockroaches, analyze FAD phylogeny, and identify a potential Δ12 FAD branch, which arose through duplication of a likely Δ9 FAD. We functionally characterize both paralogs and identify the Δ9 activity in the ancestral FAD-A1a and the Δ12 activity responsible for LA biosynthesis in FAD-A1b. Through the combination of homology modeling and site-directed mutagenesis, we pinpoint structural features possibly contributing to the distinct functions, regiospecificities, and substrate preferences of the two enzymes. We confirm the presence of both paralogs in all 36 studied species of the Blattoidea lineage (Blattidae, Lamproblattidae, Cryptocercidae, and termites) and conclude that we identified an evolutionary event important for the ecological success of termites, which took place in their cockroach ancestors roughly 160 My and remained conserved throughout termite diversification into 3,000 extant species.

## Introduction

Termites (Isoptera) are the oldest social insects and dominant animals in tropical and subtropical ecosystems. With quantitative abundance estimated to reach nearly 50 Mt of carbon, they represent roughly one-quarter of the total biomass of all terrestrial arthropods (Tuma et al. 2020). The spectacular ecological success of termites is undoubtedly due not only to their eusocial organization (Wilson 1992) but also to their dietary specialization on lignocellulose material originating from dead plant tissues. The ability to digest the recalcitrant but ubiquitous lignocellulose substrates enabled termites to become major players in carbon recycling in warmer regions of the world with important impact on the structure of landscapes and soils; termites are thus sometimes referred to as ecosystem engineers (Jouquet et al. 2011).

While being rich in energy stored in lignocellulose macromolecules, the termite diet is poor in many essential nutrients. Therefore, prior to becoming the main decomposers of cellulose materials, termites needed to evolve adaptations to cope with potential dietary constraints. The termite diet is particularly deficient in fatty acids (FAs), including polyunsaturated FAs (PUFAs), which belong, in the vast majority of animals, among essential nutrients obtained exclusively via dietary supply. PUFAs are key precursors for multiple critical biomolecules within the primary metabolism (triacylglycerols, eicosanoids, phospholipids, and others) that are used for energy storage, as components of cellular and subcellular membranes, and as signaling molecules within intracellular pathways (Pelley 2012). In insects, PUFAs have yet another important function as a part of secondary metabolism, because they serve as precursors for cuticular hydrocarbons, which protect insect bodies against desiccation and also give rise to many insect pheromones, including the greatly diversified repertoire of sex pheromones in moths (Tillman et al. 1999; Symonds and Elgar 2008).

A key step in the biosynthesis of PUFAs is the formation of linoleic acid (*Z*9,*Z*12-18:COOH, hereafter also LA) via the introduction of a second double bond in the ubiquitous monounsaturated oleic acid (*Z*9-18:COOH, OA). Animals were long thought to be incapable of LA biosynthesis and to rely purely on its dietary supply. However, the past few decades firmly established that some animal lineages can synthesize LA de novo using their own enzymatic machinery. LA production has been experimentally demonstrated in nonarthropod taxa such as nematodes or snails and slugs (Gastropoda), as well as in noninsect arthropods such as mites (Acari) (Rothstein and Götz 1968; Weinert et al. 1993; Aboshi et al. 2013; Shimizu et al. 2014). In insects, LA biosynthesis is missing in basal orders (e.g., Zygentoma, Ephemeroptera, and Odonata) and basal Polyneoptera (Dermaptera and Plecoptera). In more derived orders, de novo LA production has been recorded in almost 20 species of Orthoptera, Blattodea, Hemiptera, Hymenoptera, Neuroptera, and Coleoptera (reviewed in Malcicka et al. 2018). However, these lineages also contain species in which the LA biosynthesis has been disproved, suggesting a complex scenario of its evolution, accompanied by multiple origins and/or losses. Interestingly, LA synthesis has not been demonstrated in Lepidoptera and Diptera, two modern and very diversified insect orders representing over one-quarter of insect species richness (Grimaldi and Engel 2005), even though both orders, and especially moths (Ando et al. 2004), extensively use PUFA-derived pheromones and are able to desaturate fatty acyl substrates at various positions.

The patchy distribution of LA biosynthesis also applies for cockroaches and termites, together making up the order Blattodea. The American cockroach *Periplaneta americana* was among the first insects with confirmed LA biosynthesis (Louloudes et al. 1961), which has since been also observed in a few other cockroaches from the Blattoidea lineage, including all three studied species of termites, while being seemingly absent in the Blaberoidea clade (Bade 1964; Mauldin et al. 1972; Blomquist et al. 1982; Cripps et al. 1986; de Renobales et al. 1987; Malcicka et al. 2018). Therefore, the most parsimonious hypothesis based on the current knowledge and modern molecular phylogeny of Blattodea (Evangelista et al. 2019) would be that LA biosynthesis evolved once in basal Blattoidea, a lineage encompassing Blattidae, Lamproblattidae, and Cryptocercidae as a sister group to termites, and termites themselves.

Unsaturated FAs arise via desaturation steps catalyzed by two types of iron-containing fatty acyl desaturases, that is, soluble acyl–acyl carrier protein desaturases restricted to higher plants and structurally unrelated membrane fatty acyl-coenzyme A (CoA) desaturases (hereafter referred to as FADs) ubiquitous in endoplasmic reticulum membranes of Eukaryota (Shanklin and Cahoon 1998; López Alonso et al. 2003). FADs are oxygen- and NADH-dependent enzymes and belong to the superfamily of membrane diiron–containing enzymes, sharing a conserved tripartite histidine-rich motif, coordinating two iron atoms and four transmembrane domains as common features (Sperling et al. 2003). The current knowledge suggests that eukaryotic FADs evolved through an ancestral gene diversification of a canonical Δ9 FAD (López Alonso et al. 2003), which introduces a *Z*9 double bond to saturated stearoyl-CoA to give rise to oleoyl-CoA. OA is a universally present FA and a dominant component of the FA pool also in insects including termites (Carter et al. 1972; Stanley-Samuelson et al. 1988; Šobotník et al. 2006). Because of their crucial role in generating FA-derived pheromone diversity, insect FADs received much attention, especially in moths, beetles, and hymenopterans, with several dozen being functionally characterized and analyzed with respect to underlying gene evolution. The repertoire of insect FADs consists of multiple gene clusters (six subfamilies according to Helmkampf et al. 2015), subjected to lineage-specific expansions via duplications and neofunctionalizations on the one hand and losses on the other hand (Knipple et al. 2002; Roelofs and Rooney 2003; Helmkampf et al. 2015; Groot et al. 2016; Tupec et al. 2017). The diversity of insect FAD products results not only from different substrate specificities and stereo- and regiospecificities of individual FADs, but also from the versatility of FAD functions: some of them exhibit substrate-dependent specificities, conjugase activities, and other substrate-driven features that can be involved, for example, in the sequential synthesis of multiple double bonds in PUFAs (Moto et al. 2004; Matoušková et al. 2007; Serra et al. 2007; Wang et al. 2010; Ding et al. 2011; Buček et al. 2015).

The first insights into FAD functional determinants have been obtained using inspections of aligned sequences followed by domain swapping and/or site-directed mutagenesis experiments (Vanhercke et al. 2011; Meesapyodsuk and Qiu 2014; Buček et al. 2015; Ding et al. 2016), and recently, they have also been inferred from crystal structures of two mammalian FADs (Bai et al. 2015; Wang et al. 2015; Shen et al. 2020). In C18-specific Δ9 FADs, which are presumably ancestral to other FADs, the enzyme specificity is thought to be maintained by two major structural features: acyl register and tunnel capping (Meesapyodsuk and Qiu 2014; Bai et al. 2015; Wang et al. 2015; Wilding et al. 2017; Cai et al. 2020). In closer detail, the C1 acyl moiety of the substrate forms a hydrogen bond with the acyl register residue (e.g., tryptophan in Δ9 FADs) located near the opening of the substrate tunnel (Bai et al. 2015; Wang et al. 2015; Wilding et al. 2017; Cai et al. 2020). This binding ensures that the desaturation occurs regiospecifically at a certain distance (ΔX) from the acyl moiety. Additionally, the desaturase controls the overall length of the substrate entering the substrate tunnel (i.e., its chain length specificity) by a bulky capping residue located in the distal section of the substrate tunnel (Bai et al. 2015; Cai et al. 2020). When the capping residue appears closer to the active site, it effectively decreases the tunnel length, and as a result, the desaturase prefers shorter substrates, for example, C14 and C16 (Bai et al. 2015; Nagao et al. 2019). Alternatively, the absence of any capping residue can allow substrates as long as C26 to be desaturated (Meesapyodsuk and Qiu 2014; Cai et al. 2020). Multiple studies have tried to pinpoint the exact mutations in FADs leading to a shift in substrate specificity or regiospecificity (Vanhercke et al. 2011; Bai et al. 2015; Shi et al. 2015; Cai et al. 2020). Uncovering the precise structural feature which determines the course of substrate desaturation still remains challenging as it usually depends on multiple residues. Moreover, there are no published structures of desaturases with specificities other than Δ9 (such as Δ11, Δ12, Δ14, ω3, or ω6), which limits the search only to homologous models. However, a few examples show a profound shift in the FAD specificity due to a single residue change in the substrate tunnel (Meesapyodsuk and Qiu 2014; Buček et al. 2015, 2020; Ding et al. 2016; Cai et al. 2020).

**Fig. 1.**
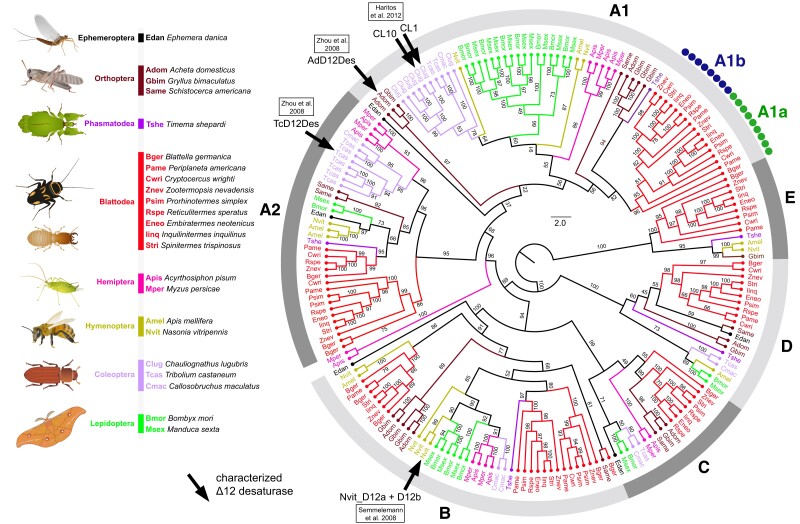
Diversity of FAD genes in Blattodea and other insects. Phylogeny of FADs inferred from 102 protein sequences of 23 insect species, including 9 Blattodea (6 termites and 3 cockroach species). Nonblattodean species were selected to include all previously identified insect Δ12 desaturases producing LA (marked with arrows). Labeling of FAD subfamilies according to Helmkampf et al. (2015). Two subclasses identified for Blattodea within the A1 subfamily are marked as A1a and A1b. The topology and branching supports were inferred using the IQ-TREE maximum likelihood algorithm with the LG+R7 model. The bootstrap values were calculated using ultrafast bootstrap approximation (UFBoot) from 1,000 replicates. Accession numbers of the analyzed FAD sequences are provided in supplementary table S1, Supplementary Material online.

The previous search for insect Δ12 FADs responsible for de novo LA formation has been motivated by both the crucial significance of LA in general metabolism and its precursor role in the production of pheromones and defensive chemicals. LA-producing Δ12 FADs have so far been identified in four species. They were first recorded in 2008 in the house cricket *Acheta domesticus* and the red flour beetle *Tribolium castaneum* (Zhou et al. 2008) and later in the soldier beetle *Chauliognathus lugubris* (Haritos et al. 2012) and the parasitic wasp *Nasonia vitripennis* (Semmelmann et al. 2019). While the former two species showed a single Δ12 FAD each, the latter two express two LA-producing paralogs within species-specific FAD expansions. Besides the potential role in primary FA metabolism, the products of some of these FAD paralogs serve as precursors for the biosynthesis of specialized secondary metabolites used for defense (*C. lugubris*) and communication (*N. vitripennis*). Most of the listed Δ12 FADs show amino acid sequence similarities with conserved Δ9 FADs and at times even retained residual Δ9 activity (Zhou et al. 2008), suggesting their origin via duplications and neofunctionalizations of the ancestral Δ9 FADs producing OA and/or palmitoleic acid. At the same time, phylogenetic analysis of insect FADs clearly indicates independent origins of Δ12 FADs in individual taxa, as Δ12 FADs may even have evolved from Δ9 FADs situated in different insect FAD subfamilies (see fig. 1).

In this study, we search for the evolutionary origin of de novo LA biosynthesis in termites. We test the hypothesis that LA biosynthesis evolved within the Blattoidea clade and that termites thus inherited this capacity from their cockroach ancestors. For this goal, we compile a repertoire of FAD homologs from 57 species of cockroaches and termites, analyze their phylogenetic patterns, identify a candidate FAD branch with potential Δ12 activity which arose through duplication of a likely Δ9 FAD within Blattoidea, and confirm the presence of the duplicated paralog in all subclades of Blattoidea (Blattidae, Lamproblattidae, Cryptocercidae, and termites). We functionally characterize both paralogous FADs using yeast expression system and identify the Δ9 activity in the ancestral FAD and Δ12 activity in its paralogous copy, giving rise to LA from OA. In parallel, using AlphaFold2-derived 3D models of the substrate tunnel and site-directed mutagenesis, we identify a putative acyl register residue needed for Δ9 activity and tunnel capping residues responsible for differential substrate selectivity of the two enzymes.

We conclude that we identified an evolutionary event potentially crucial for the dietary specialization of termites allowing them to survive on a poor lignocellulose diet, which took place roughly 160 My and remained conserved in its main functional features throughout termite evolution.

## Results

### Repertoire of Blattodean FAD Genes

In the first step, we studied the overall diversity of FAD-coding genes in a subset of three cockroach and six termite species in the context of FADs identified in 14 selected nonblattodean insects, including all those known to possess a functional LA-producing Δ12 FAD. A series of BLAST searches in public databases and our in-house sequencing data generated a set of 198 FAD sequences from the 23 species (supplementary table S1, Supplementary Material online). In the phylogenetic reconstruction (fig. 1), these sequences were distributed among six FAD subfamilies (A1, A2, and B–E) defined by Helmkampf et al. (2015). LA-producing Δ12 FADs previously identified in *A. domesticus*, *N. vitripennis*, *C. lugubris*, and *T. castaneum* were situated in three different subfamilies (A1, A2, and B), in most cases within species-specific FAD expansions, suggesting their independent evolution at low taxonomic levels. In most blattodean species, we retrieved eight to ten reliable FAD sequences per species. Only seven were identified in the termite *Embiratermes neotenicus* and as many as 12 in the cockroach *Blattella germanica*. Blattodean FADs were represented in all subfamilies as one or two monophyletic Blattodea-specific branches, each mainly containing phylogenetically organized orthologs of all or most studied species, indicating the ancient origin of individual FADs.

### Identification of a Candidate Δ12 Desaturase FAD-A1b

A closer look at the phylogeny revealed an interesting branching pattern within the FAD-A1 subfamily (fig. 1). In this subfamily, the blattodean monophylum contained two FAD sequences for all species except *B. germanica*, which was represented by the basally situated single sequence. The most parsimonious scenario of the origin of this pattern would be an ancient duplication of the ancestral FAD-A1 and subsequent conservation of its paralogous copy during diversification of blattoid cockroaches and termites. Indeed, A1 topology mostly supported this view since, for most species, each of the two putative copies was situated on one of two divergent subbranches.

**Fig. 2.**
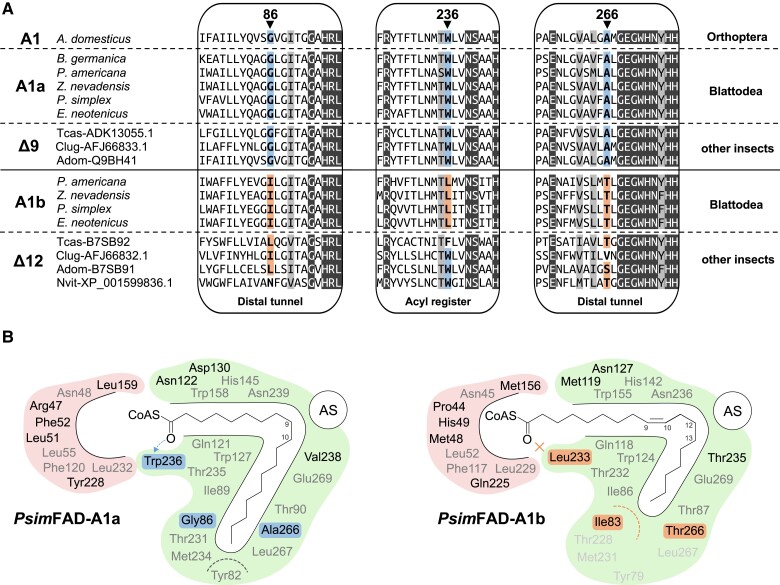
Identification of candidate Δ12 FAD in Blattodea within the FAD-A1 subfamily. (*A*) Partial alignments of protein sequences of FAD-A1a and FAD-A1b desaturases from five blattodean species compared with previously functionally characterized Δ9 and Δ12 FADs from other insects and with FAD-A1 from the cricket *A. domesticus* (numbered according to the *P. simplex* FAD-A1a sequence). Arrowheads highlight three positions in the internal region of the substrate tunnel that consistently distinguish the blattodean FAD-A1a and FAD-A1b proteins and that may have an impact on substrate specificity and desaturation regiospecificity (especially positions 86 and 236) based on previous data on FADs. Fully conserved residues are marked by dark shading and residues with similar physicochemical properties by light-gray shading. Sequences were aligned using the MUSCLE v5 algorithm. Complete protein sequences are aligned in supplementary figure S1, Supplementary Material online. (*B*) 3D model-based diagrams of the substrate tunnels of FAD-A1a and FAD-A1b in the termite *P. simplex*, fitted with their putative substrates, that is, stearoyl-CoA for the presumed Δ9 activity of FAD-A1a and oleoyl-CoA for the hypothesized Δ12 activity of FAD-A1b. Internal region of the substrate tunnel (right part of the model) and external region (left part). Gray font represents residues fully conserved in the eight studied Blattodea having FAD-A1b in figure 1, and black font denotes residues with some substitutions. Background shading marks the residues consistently substituted and highlighted in the alignment in (*A*). Hydrogen bonding to the acyl register residue Trp236 is indicated by an arrow, whereas the absence of such bonding with Leu233 is indicated by a cross. AS, active site.

The inspection of blattodean FAD-A1 amino acid sequences (figs. 2*A* and S1, Supplementary Material online) and AlphaFold2-derived 3D model of the substrate tunnel (fig. 2*B*) confirmed the hypothesized evolutionary history and split the identified sequences into two groups. All nine species, including the basal *B. germanica*, possessed one FAD copy sharing the tunnel architecture reminiscent of the known C18-specific Δ9 FADs (e.g., Cai et al. 2020), particularly the tunnel length defined by bulky Tyr82 residue (all amino acid numberings according to the sequence of *Prorhinotermes simplex*; figs. 2 and S1, Supplementary Material online) and putative acyl register mediated by Trp236. This suggests that this gene, hereafter referred to as FAD-A1a, is a Δ9 FAD and represents the ancestral A1 paralog, because the same sequence and structure features are also shared by the basal *B. germanica* FAD-A1 as well as the A1 sequence in the sister group, the cricket *A. domesticus* (figs. 1 and 2*A*).

In contrast, the paralogous A1 sequences, hereafter referred to as FAD-A1b, have several substitutions in the substrate tunnel that are shared by all nine species. Most importantly, the model indicates tunnel shortening by substituting FAD-A1a Gly86 with bulkier isoleucine residue, and the hypothesized acyl register at Trp236 is lost due to substitution with leucine. These combined substitutions could contribute to a possible shift of desaturation regiospecificity from ω9 to ω6/7, as needed for OA conversion into LA (fig. 2*A* and *B*). Additional frequent substitutions in the internal region of the substrate tunnel were Ala266Thr, Asn122Thr, and Asp130Asn. In the external region, we detected a few more frequent (Arg47Pro, Leu51Met, and Phe52His) or systematic (Leu159Met) substitutions (fig. 2*B*).

### FAD-A1a Is a Δ9 Desaturase with a Broad Range of Substrates

**Fig. 3.**
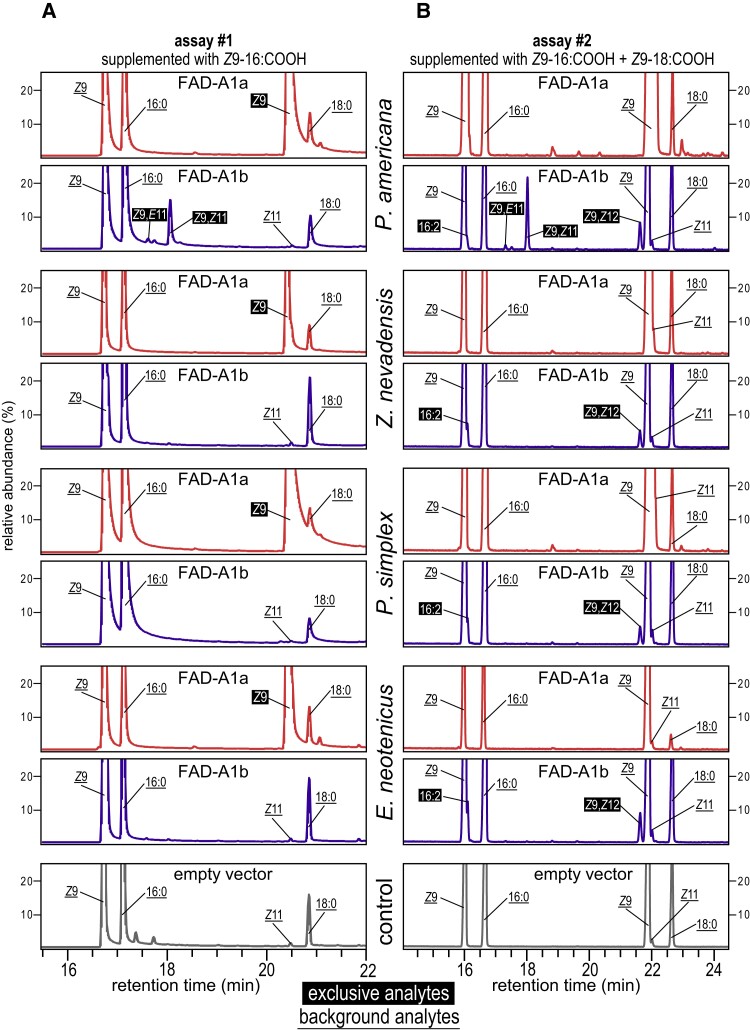
Functional characterization of FAD-A1a and FAD-A1b in the cockroach *P. americana* and three termite species from three different families. Figure shows a C16–C18 portion of GC chromatograms of yeast lipid extracts obtained after 4 days of cultivation. Empty vector serves as a negative control. (*A*) Assay #1 with *Z*9-16:COOH supplementation. (*B*) Assay #2 with *Z*9-16:COOH + *Z*9-18:COOH supplementation. Yeasts (*S. cerevisiae* strain *elo1*Δ*ole1*Δ) were transformed with pYEXTHS-BN–derived constructs containing full coding sequences of the studied insect FADs. Prior to GC analyses, the extracts were transesterified to convert FAs into FAMEs. Full chromatograms are given in supplementary figures S2 and S3, Supplementary Material online, and identifications of individual FAMEs were supported by supplementary figure S4, Supplementary Material online.

Having identified FAD candidates for the ancestral Δ9 and derived Δ12 desaturase function, we decided to functionally characterize FAD-A1a and FAD-A1b from the cockroach *P. americana* and from three species of termites using heterologous expression in desaturase-deficient yeast, supplemented with monounsaturated FAs to ensure their survival. The selection of termite species represented three families and covered the phylogenetic diversity of Isoptera: *Zootermopsis nevadensis* (Archotermopsidae), *P. simplex* (Rhinotermitidae), and *E. neotenicus* (Termitidae). Results of the main assays #1 and #2 (supplementation with *Z*9-16:COOH and *Z*9-16:COOH + *Z*9-18:COOH, respectively) are shown in figure 3 as C16–C18 portions of gas chromatograms of transesterified yeast lipid extracts; full chromatograms are provided in supplementary figures S2 and S3, Supplementary Material online. FAD-A1a from all studied species showed a Δ9 desaturase activity on the native stearic acid substrate, manifested by the occurrence of large exclusive peaks of *Z*9-18:COOMe (OA methyl ester) in assay #1 (fig. 3*A*) and a marked increase in *Z*9-18:COOMe quantity apparent in assay #2, adding to the OA that has been supplemented to the cultures (fig. 3*B*). Identifications of reported analytes are supported by supplementary figure S4, Supplementary Material online.

**Fig. 4.**
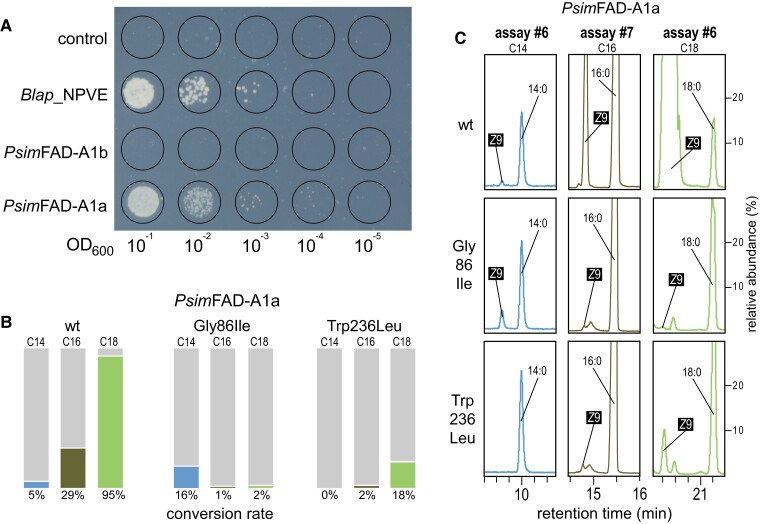
Function of FAD-A1a. (*A*) Rescue experiment comparing FAD-A1a and FAD-A1b. The photograph shows an agar plate after 4-day growth of *S. cerevisiae elo*1Δ*ole*1Δ strains expressing negative control (“control,” i.e., empty vector), positive control (Blap_NPVE, Δ9 FAD from *B. lapidarius*), and *P. simplex* FAD-A1a and FAD-A1b. OD_600_ indicates optical density of the starting culture at a wavelength of 600 nm. (*B*) Comparison of conversion rates of saturated FAs (myristic, palmitic, and stearic) into *Z*9-14:COOH, *Z*9-16:COOH, and *Z*9-18:COOH, respectively, using yeast expressing wild-type (wt) *Psim*FAD-A1a and its Gly86Ile and Trp236Leu mutants. Cultures were supplemented with *Z*9-16:COOH (assay #6) or OA (assay #7), cultured for 4 days, and the lipid extracts transesterified to convert FAs into FAMEs. Conversion rates were calculated from two replicates for each strain, and raw data are shown in supplementary table S2, Supplementary Material online. (*C*) C14, C16, and C18 portions of GC chromatograms of extracts from yeasts expressing wt *Psim*FAD-A1a and its mutants with a substitution in the distal substrate tunnel (Gly86Ile, tunnel shortening) and acyl register (Trp236Leu, loss of register at this location). Cultures were supplemented with *Z*9-16:COOH, cultured for 4 days, and the extracts transesterified to convert FAs into FAMEs.

FAD-A1a showed broad substrate preferences, because its desaturation activity was also observed for palmitic acid, giving rise to *Z*9-16:COOH, and myristic acid, leading to the formation of *Z*9-C14:COOH. Results of assays #1–3 suggested the substrate preferences to be C18 > C16 > C14, with conversion rates of 95%, 29%, and 5%, respectively (figs. 4*B*, 4*C*, S2, S3, and S5, Supplementary Material online). Likewise, FAD-A1a showed catalytic activity also with saturated FAs longer than C18 since we observed exclusive peaks of monounsaturated fatty acyl methyl esters (FAMEs) having 20, 22, and 24 carbon atoms (supplementary fig. S2, Supplementary Material online). The former was identified as *Z*9-20:COOMe, whereas in the latter two, the double bond position could not be identified. The substrate preferences were ranked as C18 > C20 > C22 > C24.

### FAD-A1a Rescues the Desaturase-Deficient Yeast Cultures

To address the effect of Δ9 desaturation activity of FAD-A1a on the survival of the desaturase-deficient *elo*1Δ*ole*1Δ yeast strain, we performed a rescue experiment with *P. simplex* FAD-A1a and FAD-A1b, the negative control, and the positive control (Δ9 FAD from the bumble bee *Bombus lapidarius*, Blap_NPVE). The results, shown in figure 4*A*, clearly prove the ability of FAD-A1a and Blap_NPVE to rescue the cultures deficient in *Z*9-unsaturated FAs, in contrast to the negative control and FAD-A1b cultures. This indicates that FAD-A1a efficiently supplies OA and other *Z*9 unsaturated FAs to the cultures, and that FAD-A1b has a different function.

### Mutation of Gly86 (FAD-A1a) to Ile86 (FAD-A1b) Shortens the Substrate Tunnel Length and Shifts the Desaturation Closer to ω-End of the Substrate

To address the predicted impact of the substrate tunnel capping residues on the differential function of FAD-A1a and FAD-A1b, we performed functional assays #6 and #7 comparing the activity of wild-type FAD-A1a from *P. simplex* with its Gly86Ile mutant, that is, a substitution systematically occurring in FAD-A1b (fig. 4*B*, 4*C*, and supplementary table S2, Supplementary Material online). The substitution of glycine residue with the bulky isoleucine residue resulted in a dramatic drop in conversion rates of stearoyl and palmitoyl chains into corresponding *Z*9 fatty acyls (95% to 2% and 29% to 1%, respectively). By contrast, the conversion of myristoyl chain into myristoleoyl chain (*Z*9-14:) increased from 5% to 16%. This suggests that Ile86 effectively shortens the substrate tunnel, and in combination with the presumed substrate-binding residues (namely, the acyl register Trp236), it limits the binding of longer fatty acyls (C16 and C18), while being preferentially accessible to shorter substrate chain lengths. Such spatial limitation then leads to a shift in the position of the ω-end of the accepted substrate closer to the active site, that is, from ω-9 optimized for stearoyl substrate in wild-type FAD-A1a to ω-5 (myristoyl) in FAD-A1a-86Ile. This is in line with the prerequisite shift in the desaturation position of C18 substrate from ω-9 in FAD-A1a to ω-6 in FAD-A1b.

### Trp236 in FAD-A1a Is Responsible for Its High Δ9 Activity

In another mutagenesis experiment, we tried to verify our model predictions attributing to Trp236 the role of acyl register residue, presumably responsible for the proper localization of the C_9_–C_10_ bond respective to the active site, giving rise to the observed highly specific Δ9 activity of FAD-A1a. A comparison of wild-type *Psim*FAD-A1a with its Trp236Leu mutant revealed a major decrease in Δ9 desaturation activity, manifested by the drop in conversion rates of C14, C16, and C18 saturated substrates to *Z*9 homologs, that is, from 5% to 0, from 29% to 2%, and from 95% to 18%, respectively. This clearly shows that Trp236 is crucial for the high efficiency of wild-type FADA1a, especially with its preferred C18 substrate.

### Termite FAD-A1b Is a Δ12 Desaturase Specialized in the Production of LA from OA

FAD-A1b showed a different regiospecificity and substrate preferences from FAD-A1a. In cultures supplemented with OA, FAD-A1b of all four studied blattodean species acted as a Δ12 FAD and converted OA into LA, as evidenced by the presence of an abundant peak of *Z*9,*Z*12-18:COOMe (figs. 3*B*, S3, S5, and S6, Supplementary Material online). We did not observe any other C18 unsaturated product. Thus, FAD-A1b was revealed to be the LA-producing enzyme predicted by our 3D model.

When offered *Z*9-16:COOH, all four studied FAD-A1b enzymes produced a trace peak of a 16:2 FAME, whose double bond localizations could not be assigned due to very low amounts of the analyte (figs. 3*B* and S3, Supplementary Material online). Interestingly, while this trace compound was the only C16 desaturation product in the three termite species, *Pame*FAD-A1a from *P. americana* produced two additional C16 peaks, found in all out of the five independent cultivations. These were identified as FAMEs of two stereoisomers of a 9,11-diunsaturated (conjugated) 16:COOH, that is, *Z*9,*E*11-16:COOMe (minor peak) and *Z*9,*Z*11-16:COOMe (major peak) (figs. 3*A*, 3*B*, S2–S4, Supplementary Material online). The two isomers arose via Δ11 desaturation of *Z*9-16:COOH, as evidenced by their absence in cultures lacking this precursor (assay #4, supplementary fig. S6, Supplementary Material online).

We did not detect any desaturation product across the range of the naturally present saturated FAs, suggesting that FAD-A1b requires monounsaturated substrates (figs. 3, S2, S3, S5, and S6, Supplementary Material online), which is in line with the observed low viability of FAD-A1b–containing yeasts in a rescue experiment without the supplementation with unsaturated FAs (fig. 4*A*).

Taken together, these observations indicate that FAD-A1b evolved a relatively strict substrate specificity and regioselectivity for OA conversion into LA in termites while having a broader range of possible substrates and products in the cockroach *P. americana* by effectively accepting also C16 monounsaturated substrate (*Z*9-16:COOH).

### Additional Unsaturated Products Due to Yeast Elongase Activities

In addition to the desaturation products of FAD-A1a and FAD-A1b, we observed a range of small peaks of unsaturated FAMEs corresponding to elongation products of yeast FA elongases. They had a double bond at *Z*11 (sometimes even *Z*13) and arose from *Z*9 precursors (figs. 3, S2–S4, Supplementary Material online), as confirmed in assay #5 using deuterium-labeled *Z*9-18:COOH tracer and yielding labeled *Z*11-20:COOMe and *Z*13-22:COOMe (supplementary fig. S7, Supplementary Material online). This indicates the activity of yeast elongase 2 and/or 3, since elongase 1 has been knocked out in the used strain.

### Gene, Transcript, and Protein Structure Confirm the Common Evolutionary Origin of FAD-A1a and FAD-A1b

**Fig. 5.**
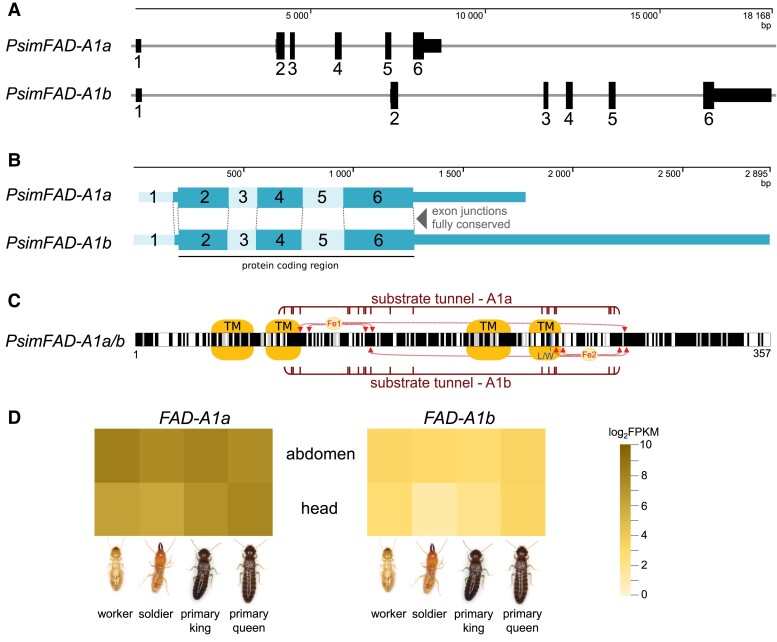
Gene, transcript, and protein structures and expression patterns of *FAD-A1a* and *FAD-A1b* in the termite *P. simplex*. (*A*) Gene structures of *FAD-A1a* and *FAD-A1b* in *P. simplex*. 3′ and 5′ untranslated regions are represented by half-size boxes. (*B*) Transcript structures of *FAD-A1a* and *FAD-A1b* in *P. simplex* highlighting fully conserved exon junctions. (*C*) Protein structures of FAD-A1a and FAD-A1b in *P. simplex* represented by an alignment of FAD-A1a and FAD-A1b. Black blocks mark fully conserved residues and gray blocks mark functionally similar residues. Transmembrane domains are shown TM boxes, cofactor binding sites for two Fe cations are marked with arrows, L/W shows the position corresponding to substrate tunnel residue Trp236 in FAD-A1a and Leu233 in FAD-A1b, and the regions forming the substrate tunnel are delimited by horizontal lines. (*D*) Heat map shows transcript abundances of *PsimFAD-A1a* and *PsimFAD-A1b* estimated from RNA-Seq experiments in two body parts (head and abdomen with removed digestive tube) and four caste phenotypes (workers, soldiers, primary kings, and primary queens). The transcript abundances are shown as log_2_FPKM values obtained as average FKPM from two (primary kings) or three (all other castes) individuals. Raw data are shown in supplementary table S3, Supplementary Material online.

We studied the gene, transcript, and protein structure of *FAD-A1a* and *FAD-A1b* using the genomic assembly of the termite *P. simplex* (fig. 5*A*–*C*). We identified two genomic loci corresponding to *FAD-A1a* and *FAD-A1b*, respectively. Both genes consist of six exons, five of which are protein coding and share highly similar structures with a high level of homology and fully conserved exon junctions. Likewise, the protein structures of FAD-A1a and FAD-A1b are highly homologous and contain all functionally important FAD domains. These observations independently confirm the common evolutionary origin of *FAD-A1a* and *FAD-A1b* and their paralogous relationship.

### *FAD-A1a* and *FAD-A1b* Show a Ubiquitous Expression in Termite Castes and Tissues Suggesting Their Participation in Primary Metabolism

The comparison of transcript abundances of *FAD-A1a* and *FAD-A1b* estimated from RNA-Seq analysis in the termite *P. simplex* revealed a ubiquitous expression of both genes in all four studied caste phenotypes (workers, soldiers, primary kings, and primary queens) and both studied tissues (head and abdomen with removed digestive tube). As shown in figure 5*D* and supplementary table S3, Supplementary Material online, *FAD-A1a* had consistently higher expression than *FAD-A1b*, roughly by one order of magnitude. In most castes, expression of both genes was slightly higher in abdominal tissues than in heads, and we did not observe any significantly caste-biased upregulation in either of the two genes. Altogether, these results show that *FAD-A1a* and *FAD-A1b* expressions are not restricted to any particular caste or organ, suggesting the participation of both genes in primary FA metabolism rather than a derived role in secondary metabolism, such as pheromone or defensive compound biosynthesis.

### *FAD-A1b* Arose via Duplication of *FAD-A1a* and Remained Conserved over 160 My

**Fig. 6.**
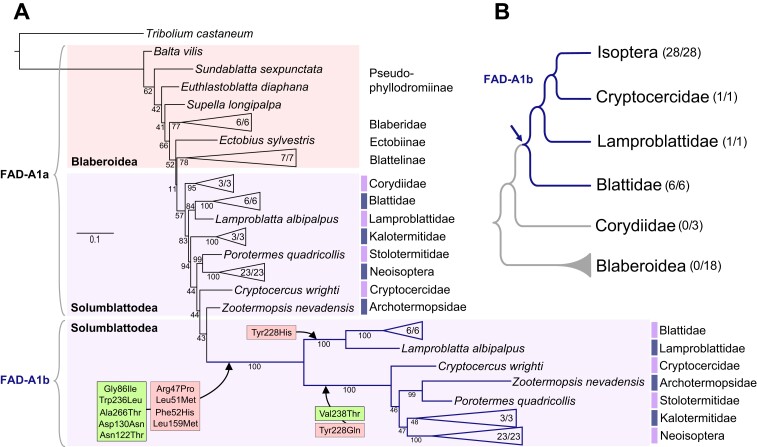
Origin of FAD-A1b via ancient duplication of FAD-A1a in basal Blattoidea. (*A*) Detailed phylogeny of FAD-A1 sequences identified in 57 Blattodea (28 termites and 29 cockroach species) and *T. castaneum* as outgroup. The numbers in condensed branches indicate the numbers of species in which the appropriate ortholog was identified/total numbers of species analyzed. Taxonomy is used according to Evangelista et al. (2019) for cockroaches and Buček et al. (2019) for termites. The topology and branching supports were inferred using the IQ-TREE maximum likelihood algorithm with the LG+R7 model. The bootstrap values were calculated using UFBoot from 1,000 replicates. Full version of the tree is given in supplementary figure S8, Supplementary Material online, and the list of studied species and accession numbers of the analyzed FAD sequences are provided in supplementary table S4, Supplementary Material online. Boxes show systematically or frequently occurring amino acid substitutions inside the internal region (green) and the external region (pink, see fig. 2*B* for a diagram) of the A1b substrate tunnel that accompanied FAD-A1b evolution. Amino acids are numbered according to FAD-A1a of *P. simplex* (fig. 2 and supplementary table S1, Supplementary Material online). (*B*) Scheme of the evolutionary origin of FAD-A1b via duplication of FAD-A1a mapped on simplified phylogenetic tree of Blattodea according to Evangelista et al. (2019). Arrow indicates the first occurrence of FAD-A1b, with its subsequent distribution marked in blue. The numbers in parentheses indicate the numbers of species in which the appropriate ortholog was identified/total numbers of species analyzed.

Following the discovery of the Δ12 FAD responsible for LA biosynthesis in termites and cockroaches, we decided to map in detail the suggested pattern of *FAD-A1b* evolution via duplication of *FAD-A1a*. We performed phylogenetic reconstruction including FAD-A1 protein sequences from 57 Blattodea, that is, 28 termite species from 7 families and 29 cockroach species from both Blaberoidea and Solumblattodea clades, with the beetle *T. castaneum* as an outgroup (supplementary table S4, Supplementary Material online). The resulting phylogenetic tree, depicted in figure 6*A*, unambiguously confirmed the hypothesized evolutionary scenario. FAD-A1a sequences were retrieved in all 57 included blattodean species, whereas the FAD-A1b sequences were found exclusively in the monophyletic branch of Blattoidea within Solumblattodea, encompassing Blattidae, Lamproblattidae, Cryptocercidae, and termites. It was present in all 36 Blattoidea species, including all 28 termites, while being absent in all three species from the sister family Corydiidae and the 18 studied Blaberoidea. A closer look at the protein alignments revealed that the two residues experimentally confirmed as important for Δ9 activity of FAD-A1a are systematically substituted during FAD-A1b evolution across Blattoidea (97% for Gly86Ile and 100% for Trp236Leu, supplementary table S5, Supplementary Material online).

Additional systematically or frequently occurring substitutions within A1b substrate tunnel are Asp130Asn (100%), Ala266Thr (97%), Asn122Thr (86%), and Val238Thr (75%) (figs. 6*A*, *6B*, and S1, Supplementary Material online, supplementary table S5, Supplementary Material online). In the external region of the substrate tunnel (fig. 2*B*), we identified yet a few other substitutions distinguishing FAD-A1b from A1a, that is, Phe52His (100%), Leu159Met (94%), Arg47Pro (88%), and Leu51Met (73%). Tyr228 from FAD-A1a was always substituted, either with glutamine (78%) or histidine (19%). What are their contributions to the distinct specificities of the two enzymes remains unknown for the moment.

Tangible functional differences between FAD-A1b of termites and that of the cockroach *P. americana* prompted the question what the *Pame*FAD-A1b sequence features are that may be responsible for this difference, and whether they represent an isolated peculiarity of *P. americana* or a common characteristic of Blattidae or Blattidae + Lamproblattidae that would reflect the evolutionary distance between these cockroach families and the monophylum Cryptocercidae + termites. Indeed, the FAD-A1b protein phylogeny revealed sequence differences between termites + *Cryptocercus* and the remainder of cockroaches, manifested by the long branch separating termites + Cryptocercidae, indicating that termite ancestors have acquired a considerable number of sequence modifications after their separation from Lamproblattidae (fig. 6*A*). A detailed analysis of the amino acid sequences of the FAD-A1b substrate tunnel supports this view and splits the studied species into two groups, that is, Blattidae + Lamproblattidae versus all termites + their sister cockroach genus *Cryptocercus*. All Blattidae + Lamproblattidae possess Val238 residue inside the tunnel cavity, inherited from FAD-A1a, while in termites + *Cryptocercus*, this amino acid is substituted with threonine (27 out of 29 species). In the external region of the substrate tunnel, all Blattidae + Lamproblattidae have Tyr228 from FAD-A1a substituted with histidine, while termites + *Cryptocercus* with glutamine (28 out of 29) (fig. 6*A*). A summary of all substitutions in the substrate tunnel is shown in Supplementary table S5 online.

All these observations brought us to the conclusion that FAD-A1b arose via duplication in the common ancestor of Blattoidea after the branching of Corydiidae (fig. 6*B*), and it remained conserved with respect to potentially functionally important residues over the 160 My of Blattoidea diversification (according to the dating estimates by Evangelista et al. 2019) while showing additional evolutionary features separating cockroach families from termites.

## Discussion

In the present study, we attempted to reconstruct the evolutionary history of FAD genes underlying the ability of termites (Isoptera) to convert OA into LA, which is crucial for their survival while being scarce in their specialized lignocellulose diet. In the quest for the responsible Δ12 FAD, we identified a candidate duplication within the FAD-A1 subfamily. The ancestral gene, FAD-A1a, is present in all studied species of Blattodea (29 cockroaches + 28 termites) as well as in other representatives of Polyneoptera and belongs among Δ9 FADs as judged from functionally important residues of the substrate tunnel. In contrast, the newly evolved FAD-A1b is restricted to the monophyletic clade Blattoidea, encompassing Blattidae, Lamproblattidae, Cryptocercidae, and termites, while being absent in all other 21 studied cockroaches from the sister lineages Corydiidae and Blaberoidea. FAD-A1b was distributed in a one-to-one orthologous pattern in all 36 studied representatives of Blattoidea and revealed conserved modifications in the substrate tunnel that we predicted to allow for Δ12 desaturase activities. A common origin of FAD-A1a and FAD-A1b was also apparent from their gene structures mapped on the genome assembly of the termite *P. simplex*, in which both paralogs shared a high level of homology and full conservation of exon–intron boundaries.

Functional characterization of FAD-A1a and FAD-A1b originating from the cockroach *P. americana* and three termite species from different phylogenetic positions using desaturase-deficient yeasts confirmed the *in silico* predicted functions of both enzymes. FAD-A1a is a Δ9 FAD introducing a *Z*9 double bond into a broad range of saturated FAs (C14–C24), with preference for stearic acid. In contrast, FAD-A1b acts as a Δ12 FAD converting OA into LA and has a relatively strict substrate specificity for OA in termites, while acting as a potentially multifunctional enzyme in the cockroach *P. americana* by also producing substantial quantities of conjugated FAs *Z*9,*E*11-16:COOH and *Z*9,*Z*11-16:COOH from *Z*9-C16:COOH. Whether these FAs are native to *P. americana* and what would be their biological significance are unknown. All these observations bring us to conclude that the ability of extant termites to synthesize LA evolved together with FAD-A1b roughly 160 My in the common ancestor of Blattoidea, was inherited by termites, and remained conserved across 140 My of their evolution and diversification into 3,000 extant termite species (Krishna et al. 2013; Buček et al. 2019; Evangelista et al. 2019). The universal presence of complete FAD-A1b transcripts, high level of conservation of functionally important amino acid residues across the phylogenetic diversity of termites, and ubiquitous expression in different castes and body parts invite us to speculate that FAD-A1b has a significant role in termite primary metabolism.

Historical focus on insect FADs involved in the biosynthesis of FA-derived pheromones has led to functional characterizations and evolutionary analysis of multiple tens of FADs with various substrate specificities and stereo- and regiospecificities, especially in numerous lepidopterans and a few hymenopteran (Knipple et al. 2002; Roelofs and Rooney 2003; Helmkampf et al. 2015; Groot et al. 2016) and dipteran species (Dallerac et al. 2000; Legendre et al. 2008; Fang et al. 2009). In addition to gaining important insights into pheromone evolution, this search was motivated by the applied potential in controlling moth pest species and biotechnological applications (Tupec et al. 2017). The amassed body of information contrasts with the scant knowledge on FADs in other insect taxa and on FADs involved in primary metabolism leading to the formation of PUFA as crucial building blocks for a variety of biomolecules. It is also the case of LA-producing Δ12 FADs, which have only been characterized in four species, in spite of the complex pattern of their repeated independent evolution from the ancestral Δ9 FADs, suggested by the few available identifications (Zhou et al. 2008; Haritos et al. 2012; Semmelmann et al. 2019) and knowledge on LA biosynthesis distribution across Insecta (Malcicka et al. 2018). These reports indicate a notable plasticity in LA biosynthesis evolution and de novo Δ12 FAD occurrence at low taxonomic levels of individual species or genera, even though this image may be biased by low sampling in the previous studies. In contrast, using extensive mining of termite and cockroach sequences, we report here the origin of a Δ12 FAD via an ancient Δ9 FAD duplication and neofunctionalization taking place deep in the history of Blattodea and its conservation over a long evolutionary period with a single paralog per species and with conserved sequence patterns. The distribution of FAD-A1b in cockroaches and termites corroborates the previous observations on blattodean LA biosynthetic capacities restricted to Blattoidea species (Louloudes et al. 1961; Bade 1964; Mauldin et al. 1972; Blomquist et al. 1982; Cripps et al. 1986; de Renobales et al. 1987; Malcicka et al. 2018) and thus confirms our initial hypothesis on single LA biosynthesis origin in this clade.

Besides the participation in primary metabolism, the de novo biosynthesis of LA may also be important for secondary metabolism. Termites, as social insects, extensively use pheromone communication to coordinate the social tasks in their colonies. Some of these pheromones are derived from unsaturated FAs, including PUFAs. It is especially the case for trail-following pheromones, used to mark the foraging trails, and sex-pairing pheromones, allowing the future kings to localize the future queen during the dispersal flights. Chemical diversity of both of these pheromone types is dominated by C12 fatty alcohols *Z*3-dodecenol, *Z*3,*Z*6-dodecadienol, and *Z*3,*Z*6,*E*8-dodecatrienol, which are used as pheromones in virtually all species of the derived clade Kalotermitidae + Neoisoptera, encompassing more than 98% of extant termite species (Bordereau and Pasteels 2011; Krishna et al. 2013). Though their biosynthesis is not yet described, they are likely to originate from the ubiquitous FAs OA and LA and proceed through three cycles of β-oxidation followed by the reduction to corresponding alcohols by fatty acyl-CoA reductases, a transformation order analogous to the origin of FA-derived alcohol pheromones in other insects (Lassance et al. 2010; Lienard et al. 2010; Jurenka et al. 2017; Tupec et al. 2019). The resulting pheromones are then expected to be stored in the form of wax esters prior to their release as free alcohols (Tokoro et al. 1990, 1992; Jirošová et al. 2016; Kyjaková et al. 2017). The availability of LA independently of dietary sources may facilitate the production of these pheromones. While the expected biosynthesis of the former two alcohols appears as relatively trivial, the formation of the latter would require an additional stereospecific desaturation at ω4 position of LA (or its shortened homolog) to give rise to the unusual 3*Z*,6*Z*,8*E* double bond topology of the final pheromone. The repertoire of FAD sequences assembled in the present study may be an initial step in the search of the FAD responsible for the introduction of this third double giving rise to the unique structure of *Z*3,*Z*6,*E*8-dodecatrienol, the most frequent termite pheromone, known to be naturally produced only by termites and the wood-degrading basidiomycete fungus *Gloeophyllum trabeum* (Esenther et al. 1961; Matsumura et al. 1968).

**Fig. 7.**
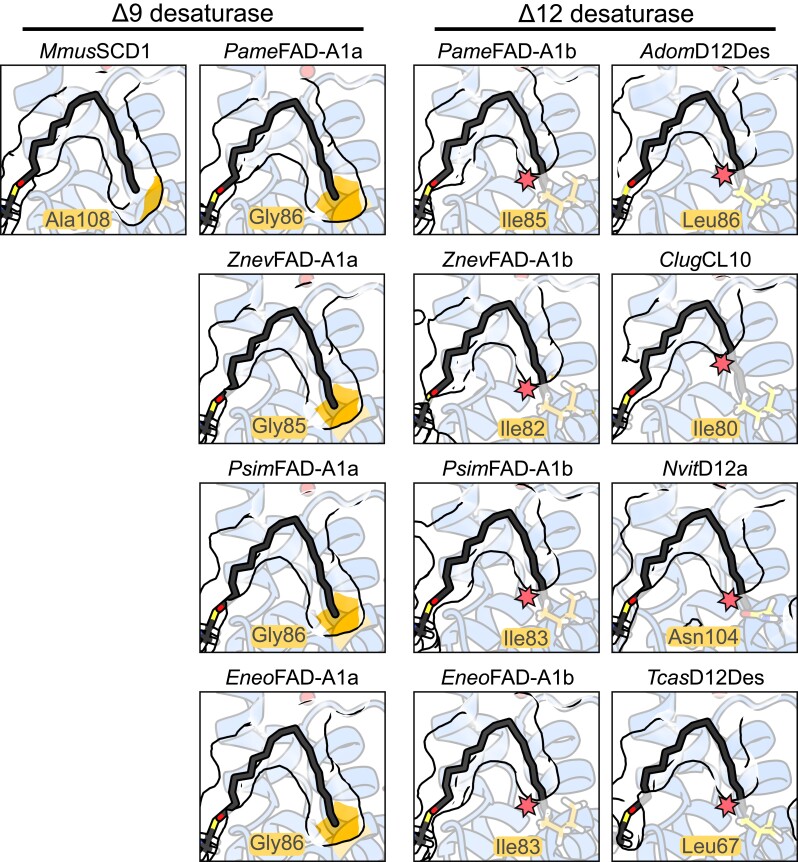
Substrate tunnels of Δ9 and Δ12 FADs. Cross-section views of the substrate tunnels in blattodean FAD-A1a and FAD-A1b and previously functionally characterized FADs from mouse (*Mmus*), cricket (*Adom*), parasitoid wasp (*Nvit*), and beetles (*Clug* and *Tcas*), illustrating the different tunnel capping architectures in Δ9- and Δ12-specific enzymes. The model of mouse SCD1 is based on its X-ray structure (Bai et al. 2015), while the rest of the models were predicted by AlphaFold2 (Mirdita et al. 2022). The active site is located at the top of each image (the ball indicating one of the metal ions). Model of stearoyl-CoA (a substrate of canonical Δ9 FADs) is fitted in all tunnels based on the structural alignment with mouse SCD1. The residues homologous to the tunnel capping residues inferred in blattodean FAD-A1b (e.g., Ile83 in *Psim*FAD-A1*b*) are represented as sticks. The star indicates a potential steric clash of stearoyl-CoA (if bound in Δ9 FAD-specific manner) with the tunnel capping residue in Δ12 FADs.

Our functional characterizations of cockroach and termite FADs point at the significance of structural aspects of the desaturase substrate tunnel. We used the insights obtained by previous studies on animal FAD structures and sequence features (e.g., Bai et al. 2015; Wang et al. 2015; Wilding et al. 2017; Nagao et al. 2019), summarized in supplementary figure S9, Supplementary Material online, to predict two possible mechanistic relationships for Δ9 and Δ12 functions. First, we verified the hypothesized role of the Trp236 residue in substrate binding specific to Δ9 function. This residue has been recorded at the opening of the substrate tunnel in multiple Δ9 FADs while being absent in some FADs of other specificities (e.g., Wilding et al. 2017). It is presumed to stabilize the thioester moiety of the substrate at a defined distance from the histidine-rich active site via hydrogen bonding. Indeed, we showed here that this residue is of great importance, because its absence dramatically decreases the Δ9 activity, yet it does not render the enzyme inactive. Notably, certain lowering of Δ9 activity was reported in a long chain–specific mutant of mouse SCD1 when its acyl register Trp258 was changed to phenylalanine or tyrosine residue, while the register mutants with other residues, including leucine, were completely inactive (Cai et al. 2020). This suggests that the acyl register is not vital for Δ9 regiospecificity of FADs, but it rather adds to the overall substrate binding mediated by CoA-binding residues in the external region of the substrate tunnel (fig. 2*B*) and on the surface of the enzyme. In all of our studied cockroach and termite FAD-A1b Δ12 desaturases, Trp236 register is lost, which may contribute to its differential function compared with FAD-A1a. However, the loss of this Δ9-specific acyl register does not appear to be universal and indicative for all Δ12 enzymes, since in some of them, it remains conserved (see fig. 2*A*). In fact, the genuine acyl register in Δ12 FADs might be significantly shifted out of the substrate tunnel in order to compensate for the shorter substrate tunnel length in these enzymes compared with the ancestral Δ9 FADs (fig. 7). Such a Δ12-specific acyl register has not yet been identified.

Second, based on previous experiments and modeling studies suggesting substrate preference shifts resulting from substrate tunnel capping (e.g., Meesapyodsuk and Qiu 2014; Cai et al. 2020), we aimed at testing the possible role of the shorter substrate tunnel observed in FAD-A1b compared with FAD-A1a. The capping residues should determine the length of accepted substrates and localization of the double bond introduction relative to the substrate ω-end. Shortening of the FAD-A1a substrate tunnel length using the substitution consistently appearing in FAD-A1b really leads to exclusion of longer substrates (C16 and C18) in favor of shorter fatty acyls (C14), thus resulting in the shift from the dominant ω-9 to ω-5 desaturation; such a shift towards the ω-end is needed for the Δ12 activity of FAD-A1b. We compared this observation with the models of Δ12 FADs previously characterized in insects and found a similar distal tunnel architecture, as evidenced in figure 7. While the tunnel capping mechanism is shared by the independently evolved insect Δ12 FADs, the overall substrate-binding pattern of Δ12 desaturases in general remains to be elucidated and will require complex mutagenesis experiments and/or structure determination. Nevertheless, the pattern of substrate tunnel capping may be used for the preliminary prediction of non-Δ9 functions.

In light of the partial functional differences between FAD-A1b in cockroaches on the one hand and termites + their sister genus *Cryptocercus* on the other hand, we identified a few sequence differences characteristic for the cockroach and termite FAD-A1b variants. What is the mechanistic role of the pinpointed differences remains unknown, as well as whether the FAD-A1b products additional to LA in *P. americana* (conjugated C16 FAs) are of adaptive significance. Even so, the ability to convert OA into LA is shared by both FAD-A1b variants and in both of them, the capacity to accept saturated substrates has been lost, as shown by both the functional assays and rescue experiments. Loss of the original desaturation function along the evolution from Δ9 to Δ12 FADs is reported in some insect desaturases, whereas in some others, the Δ9 catalytic function on saturated substrates has been in part preserved (Zhou et al. 2008).

Dietary specialization of termites and their sister group Cryptocercidae on lignocellulose substrates has been preceded by the acquisition of multiple metabolic adaptations facilitating the cellulose digestion and compensating for the deficiency in various nutrients. The establishment of obligatory symbiosis with cellulose-degrading intestinal flagellates was among the most important ones (Brune 2014), together with the acquisition of nitrogen-fixing bacteria, allowing for compensation of the low nitrogen content in a cellulose diet by fixation of atmospheric nitrogen (Breznak and Brill 1973). Since the energetic and nutrient flow between the intestinal symbionts and the termite host is primarily mediated by the microbial production of acetate (Odelson and Breznak 1983), the termite lipids are predominantly synthesized de novo from acetate in the fat bodies. Symbiont-independent LA origin in the fat body has been confirmed by previous radiotracer experiments (Mauldin et al. 1978; Blomquist et al. 1982) and is in line with our observations of ubiquitous expression of both studied genes in both the heads (lower expression) and abdomens (higher expression), both of which contain the fat body tissue. In contrast, termite dietary gains of unsaturated FAs including PUFAs are low and may essentially be due to the digestion of the symbionts themselves by the termite host. The evolution of LA biosynthesis via FAD-A1b in omnivorous termite ancestors might have been yet another critical preadaptation for the switch to the specialized lignocellulose diet and ultimately for the undisputed ecological success of termites in warmer terrestrial ecosystems.

## Materials and Methods

### Origin of Insects

Beside publicly available sequencing data, we used de novo generated transcriptomes of one lower termite species, *P. simplex* (Rhinotermitidae), and three species of higher termites (Termitidae), *E. neotenicus* (Syntermitinae), *Spinitermes trispinosus*, and *Inquilinitermes inquilinus* (both Termitinae). Multiple *P. simplex* colonies collected in Cuba and Florida (United States) between 1964 and 2009 are kept in the laboratory cultures of the authors. The authors collected the remaining three sequenced species from field colonies in the primary forest of French Guiana along the road to Petit Saut (5°04.250′N 52°58.770′W–5°04.650′N 361 53°01.360′W). Cold-anesthetized termites were dissected under stereomicroscope, the dissected tissues transferred into RNase free collection tubes, kept at 4 °C overnight in 1 mL of RNA*later* (Thermo Fisher Scientific), and transported to the laboratory in Prague at −10 °C.

### Next Generation Sequencing and Bioinformatics

Total RNA was isolated from the abdominal cavity (with removed digestive tube) and heads of 3 primary queens + 2 primary kings + 12 workers + 12 soldiers of *P. simplex*, 10 female nymphs + 10 neotenic queens of *E. neotenicus*, 16 soldiers + 16 workers of *S. trispinosus*, and 16 soldiers + 12 workers of *I. inquilinus* using the acid guanidinium thiocyanate–phenol–chloroform extraction method. Briefly, deep-frozen samples pretreated with RNA*later* were homogenized in TRI reagent solution (Thermo Fisher Scientific) using polypropylene pestles. RNA was collected from aqueous fraction after addition of chloroform and centrifugation according to manufacturer's documentation and precipitated with isopropanol (1:1), and the pellet was washed with 75% ethanol, dried at room temperature, and dissolved in 10 mm Tris-HCl buffer, pH 8.0, with 0.1 mm EDTA.

Quality control and quantification of isolated samples were performed on Nanodrop ND-1000 UV/VIS spectrophotometer (Thermo Fisher Scientific) and 1% agarose gel in Tris–EDTA–acetate buffer prestained with ethidium bromide. Preparation of poly(A)-selected strand-specific RNA libraries and Illumina sequencing was conducted by external service providers - Novogene Co. (Beijing, China), providing 414 million of 2 × 150 bp paired-end reads in *P. simplex*; Eurofins Genomics (Ebersberg, Germany), providing 220 and 178 million of 2 × 150 bp paired-end read in *S. trispinosus* and *I. inquilinus*, respectively; and GeneCore facility, EMBL (Heidelberg, Germany), providing in total 103 million of 2 × 100 bp paired-end reads in *E. neotenicus*.

Raw reads were processed with rCorrector (Song and Florea 2015), sequencing adapters and low-quality bases were trimmed using Trimmomatic v0.32 (Bolger et al. 2014), and reads originating from ribosomal RNA were filtered out based on mapping to the reference from SILVA database release 132 (Quast et al. 2012) using bowtie2 v2.3.4.1 algorithm (Langmead and Salzberg 2012). De novo transcriptome assemblies were performed using Trinity v2.1.1 (Grabherr et al. 2011) followed by prediction of coding regions with Transdecoder v5.5.0 (https://github.com/TransDecoder).

### FAD Sequence Prediction, Gene Structure, and Expression

Candidate FAD sequences were first retrieved from our in-house transcriptome assemblies of *P. simplex*, *E. neotenicus*, *S. trispinosus*, and *I. inquilinus* and publicly available assemblies of *Z. nevadensis* (GCA_000696155.1; ZooNev1.0; Terrapon et al. 2014) and *Cryptotermes secundus* (GCA_002891405.2; Csec_1.0; Harrison et al. 2018) upon multiple protein BLAST searches using a reference data set combining all sequences used in (Buček et al. 2013, 2015) and Helmkampf et al. (2015). BLAST hits were then filtered based on *e* values (≤0.001), alignment length (≥200 bp), and similarity cutoffs (≥40%), and final candidate sequences were retrieved manually in a series of multiple sequence alignments and phylogenetic analyses in SeaView v4 (Gouy et al. 2010). These candidates were later used for FAD discovery in other termites, cockroaches, and comparative insect species in Transcriptome Shotgun Assembly database (TSA, NCBI) based on homology mapping using protein BLAST.

Gene structure of PsimFAD-A1a and PsimFAD-A1b was reconstructed upon BLAST-based local alignment of predicted transcript sequence in our *P. simplex* in-house genome assembly (Koubová et al. 2021) and manual revision of exon–intron boundaries. The fragments per kilobase million (FPKM) normalized read abundances were estimated in *P. simplex* queens, kings, soldiers, and workers after bowtie2 mapping of the RNA-Seq reads on transcriptome assembly based on RNA-Seq by Expectation-Maximization (RSEM) method using the align_and_estimate_abundance.pl script from Trinity v2.1.1 package.

### Phylogenetic Tree Reconstruction

Two phylogenetic analyses of insect FADs were performed. The first one encompassed 23 species from 7 orders across the diversity of Insecta, that is, Ephemeroptera (1 species), Orthoptera (3), Phasmatodea (1), Blattodea (3 cockroaches + 6 termites), Hemiptera (2), Hymenoptera (2), Coleoptera (3), and Lepidoptera (2), including all previously reported Δ12 FAD sequences giving rise to LA (*A. domesticus*, *N. vitripennis*, *C. lugubris*, and *T. castaneum*). The complete list of species and sequence accession numbers is provided in supplementary table S1, Supplementary Material online.

The second analysis aimed at a detailed view on the evolutionary pattern of FAD-A1 branch (according to Helmkampf et al. 2015), a subfamily identified by the previous phylogeny and amino sequence analysis to contain putative Δ12 FADs evolved from an ancestral Δ9 FAD. It included 29 species of cockroaches from both Blattoidea and Blaberoidea lineages and 28 species of termites from 7 families. List of species and FAD sequences is provided in supplementary table S4, Supplementary Material online.

Sequence alignments were generated with two to four iterations of the MUSCLE algorithm (Edgar 2004). In case of the first analysis, highly variable N- and C-terminal parts were trimmed and only the relevant conserved region covering all functional domains and corresponding to alignment positions 50–329 in *P. simplex* FAD-A1b protein sequence was kept for tree building (see supplementary table S1, Supplementary Material online). Maximum likelihood trees were inferred in IQ-TREE (Nguyen et al. 2015) using LG+R7 as the best fitting substitution model, and branch supports were based on 1,000 ultrafast bootstrap replicates (Hoang et al. 2018).

### Cloning and Expression of Candidate Genes in Yeast

Functional characterization was performed for FAD-A1a and FAD-A1b from the cockroach *P. americana* (*Pame*) and three termite species, that is, *Z. nevadensis* (*Znev*, Archotermopsidae), *P. simplex* (*Psim*, Rhinotermitidae), and *E. neotenicus* (*Eneo*, Termitidae). Synthetic FAD sequences codon optimized for yeast (see supplementary table S6, Supplementary Material online) were custom cloned (GenScript) into copper ion–inducible pYEXTHS-BN vector (Holz et al. 2002), equipped with an N-terminal hexahistidine (His_6_) sequence. After verifying the cloned FAD sequences by Sanger sequencing (Eurofins Genomics) using primers 5′-AATATCATATAGAAGTCATCG and 5′-TTTGCAGCTACCACATTG, the vectors were transformed into desaturase 1- and elongase 1-deficient *Saccharomyces cerevisiae* strain *elo*1Δ*ole*1Δ (MATa *elo1:HIS3 ole1:LEU2 ade2 his3 leu2 ura3*) (Schneiter et al. 2000). The yeasts were cultured in a synthetic complete medium lacking uracil (SC−Ura), supplemented with 0.5 mm copper sulfate and 0.1% (*w*/*v*) tergitol (type NP-40, Sigma). To allow the growth of the mutant host strain lacking Δ9 desaturase, the medium was supplemented with 0.1 mm*Z*9-monounsaturated fatty acid(s) (UFA, see below). Heterologous protein expression was monitored by western blot analysis of the whole-cell extracts. First, the harvested cells were incubated for 5 min in 5% (*v*) β-mercaptoethanol and subsequently for 5 min in 0.4 m NaOH to partially disintegrate the cell walls and then treated with regular sample buffer for SDS–PAGE. The His_6_-tagged proteins were detected using mouse antipolyhistidine–HRP conjugate antibody (Sigma-Aldrich, A7058, 1:2,000 dilution) and Ultra Science Femto Western Substrate kit (Bio-Helix). As a negative control, we used a strain bearing an empty pYEXTHS-BN vector, and as positive controls, we used Blap_NPVE, a Δ9 FAD from the bumble bee *B. lapidarius* (Buček et al. 2013).

The complete protein-coding sequences (i.e., including the His_6_ tag) of newly characterized blattodean FADs were deposited to GenBank under accession numbers OP575963–OP575970.

### Site-Directed Mutagenesis

To confirm the functional significance of the amino acid residues expected to codetermine the Δ9 activity of FAD-A1a, we performed functional assay #6 comparing the activity of wild-type *Psim*FAD-A1a from *P. simplex* with that of its mutants *Psim*FAD-A1a-86Ile and *Psim*FAD-A1a-236Leu. Both mutants were generated by whole plasmid amplification (from 25 ng template pYEXTHS-BN*_Psim*FAD-A1a) using Q5 high-fidelity polymerase (New England Biolabs) and mutagenic primers listed in supplementary table S7, Supplementary Material online. The amplification was carried out for 30 s at 98 °C, followed by 3 cycles of 20 s at 98 °C, 20 s at 51 °C, and 4.25 min at 72 °C, followed by 25 cycles of 10 s at 98 °C, 20 s at 60 °C, and 4.25 min at 72 °C, and terminatedby 10 min at 72 °C. After amplification, the template was digested with *Dpn*I nuclease (New England Biolabs) at 37 °C for 3, and 10 μL of the digested mixture was transformed into *Escherichia coli* strain DH5α (Invitrogen). The mutant constructs were then amplified in bacteria, isolated using Zyppy Plasmid Miniprep kit (Zymo Research) and Sanger sequenced (Eurofins Genomics) prior to the transformation into yeast.

### Desaturase Functional Assays

Yeasts were cultured for 4 days at 30 °C in 20 mL of medium with variable UFA supplementations to cover the range of possible substrates while still allowing for yeast growth. Following cultures were prepared, differing in the supplemented UFAs and tested enzymes: *assay #1*, palmitoleic acid (Fluka), *Psim*FAD-A1a, *Psim*FAD-A1b, *Pame*FAD-A1a, *Pame*FAD-A1b, *Eneo*FAD-A1a, *Eneo*FAD-A1b, *Znev*FAD-A1a, and *Znev*FAD-A1b; *assay #2*, palmitoleic acid + OA (Sigma-Aldrich), PsimFAD-A1a, *Psim*FAD-A1b, *Pame*FAD-A1a, *Pame*FAD-A1b, *Eneo*FAD-A1a, *Eneo*FAD-A1b, *Znev*FAD-A1a, and *Znev*FAD-A1b; *assay #3*, myristic acid (Fluka) + OA, *Psim*FAD-A1a, and *Psim*FAD-A1b; *assay #4*, OA and *Pame*FAD-A1b; *assay #5*, lauric acid (Sigma-Aldrich) + (15,15,16,16,17,17,18,18,18)-*d*_9_-OA (Avanti Polar Lipids), *Psim*FAD-A1a, and *Psim*FAD-A1b; *assay #6*, palmitoleic acid, *Psim*FAD-A1a, *Psim*FAD-A1a-86Ile, and *Psim*FAD-A1a-236Leu; and *assay #7*, OA, *Psim*FAD-A1a, *Psim*FAD-A1a-86Ile, and *Psim*FAD-A1a-236Leu. After harvesting, the cells were washed once with 1% (*w*/*v*) tergitol, twice with water, and lyophilized before lipid extraction.

### Lipid Extraction and Transesterification

The lipids from the yeast cultures were extracted and transesterified as previously described (Matoušková et al. 2007; Tupec et al. 2019) with slight modifications. Briefly, the lyophilized sample was shaken vigorously with 1.2 mL of dichloromethane (DCM)/methanol 2:1 (*v*/*v*) and glass beads (0.5 mm) for 1 h. After short centrifugation to sediment the cellular debris, 1 mL of supernatant was evaporated under nitrogen, and the residue was shaken with 0.2 mL of 0.5 m sodium methoxide in methanol for 0.5 h. The mixture was neutralized by adding 0.2 mL of solution of disodium phosphate and monopotassium phosphate (0.25 m each) and 35 μL of 4 m HCl. The obtained FAMEs were extracted with 600 μL of hexane, and a drop of 0.6 m trimethylsilyldiazomethane in hexane was added to the extract, which was then analyzed by gas chromatography (GC).

### Chromatographic Analyses

For analyses of the FAME fraction, we used GC coupled with mass spectrometric detection (GC–MS) and 2D GC coupled with time-of-flight mass spectrometric detection (GC × GC–TOFMS). GC-MS analysis was run on a TRACE 1310 gas chromatograph coupled with an ISQ LT mass spectrometer with electron ionization (70 eV) and a quadrupole mass analyzer (Thermo Fisher Scientific, USA) equipped with nonpolar ZB-5 column (Phenomenex, USA; 30 m, i.d. 0.25 mm, and 0.25 μm film thickness). The split/splitless (SSL) inlet was heated to 250 °C, the oven programmed to 50 °C (1 min)–8 °C/min–320 °C (5 min) or for better chromatographic resolution to 140 °C (1 min)–4 °C/min–280 °C (2 min)–10 °C/min–320 °C (5 min).

For GC × GC–TOFMS analyses, we used Pegasus BT 4D (Leco, USA) chromatograph with liquid nitrogen thermal modulator, equipped with a combination of nonpolar Rxi-5MS (Restek, Bellefonte, PA, USA; 30 m, i.d. 0.25 mm, and 0.25 μm film thickness) and medium-polarity Rxi-17SilMS (Restek, Bellefonte, PA, USA; 1.5 m, i.d. 0.1 mm, and 0.1 μm film thickness) columns. The SSL injector was heated to 250 °C, the oven programmed to 50 °C (1 min)–8 °C/min–320 °C (5 min) with the modulator set 15 °C higher and the secondary oven 10 °C higher than the primary column. The instrument was operated either in 1D or 2D setup depending on the required peak resolution. We used electron ionization (70 eV), time-of-flight mass analyzer, at acquisition rate 15 spectra/s (1D) or 200 spectra/s (2D).

### FAME Derivatization

To identify the double bond positions in the analyzed FAMEs, we performed two types of microderivatizations, that is, conversion to dimethyloxazoline derivatives (DMOX) (Fay and Richli 1991) and methylthiolation using dimethyl disulfide (DMDS) (Dunkelblum et al. 1985). The DMOX derivatization was carried out by adding 500 µL of 2-amino-2-methylpropanol to the FAME samples, which have been rid of the original solvent (hexane) under a stream of nitrogen at room temperature. The samples were then heated with the reagent overnight at 180 °C. After cooling, the reaction mixture was dissolved in 5 mL of DCM and washed twice with 2 mL of water. The DCM solution was subsequently dried with disodium sulfate and evaporated under a stream of nitrogen at room temperature. The residue was dissolved in 200 µL of hexane and injected into the GC system.

For DMDS derivatization, 100 µL of DMDS and 5 µL of 5% (*w*/*v*) iodine in diethyl ether were added to 100 µL of FAME samples in hexane. The reaction mixture was kept at 40 °C overnight. The solution was decolorized with two drops of 5% (*w*/*v*) sodium thiosulfate solution and the products were extracted into hexane (twice 300 µL). The organic layer was redissolved in hexane and injected into the GC system.

### Rescue Experiment

We tested the potential rescuing ability of FAD-A1a and FAD-A1b in the *elo1*Δ*ole1*Δ yeast strain. To do so, a cell suspension (OD_600_ 0.1) was stepwise diluted to OD_600_ 10^−5^ and the suspensions were applied as drops to SC−Ura plates with 0.5 mm copper sulfate using a replica plater (Merck, 96-well plate 8 × 12 array); growth control plates with 0.1 mm palmitoleic acid and OA (each) and 0.1% (*w*/*v*) tergitol were also prepared. The plates were incubated for 4 days at 30 °C and subsequently photographed. The experiment was performed with FAD-A1a and FAD-A1b from *P. simplex* (*Psim*FAD-A1a and *Psim*FAD-A1b), a positive control (Blap_NPVE), and a negative control (yeast transformed with an empty plasmid).

### Structure Prediction

The structural models of FAD-A1a and FAD-A1b from *P. americana*, *Z. nevadensis*, *P. simplex*, and *E. neotenicus* and of Δ12 FADs from *A. domesticus* (UniProt B7SB91), *T. castaneum* (B7SB92), *C. lugubris* (K7PD28), and *N. vitripennis* (A0A7M7GAP9) were predicted using ColabFold (Jumper et al. 2021; Mirdita et al. 2022) with default parameters (pdb70 template mode). The PDB files with the models used in this study are available in supplementary archive 1, Supplementary Material online. The predicted FAD models were manually trimmed of low-pLDDT terminal regions and aligned to a complex of mouse SCD1 with stearoyl-CoA (PDB code 4YMK) in PyMOL Viewer (Schrodinger). The individual substrate tunnels were subsequently inspected using UCSF ChimeraX 1.4 (Pettersen et al. 2021).

## Supplementary Material

msad087_Supplementary_Data

## Data Availability

Data generated or analyzed during this study are included in the main article file, supplementary information file, Supplementary Material online, and in supplementary archive, Supplementary Material online. Full read sequences from RNA-Seq experiments for *P. simplex*, *E. neotenicus*, *S. trispinosus*, and *I. inquilinus* are provided in a form of multiple SRA archives under NCBI BioProject accession PRJNA918850, and complete gene-coding sequences of *P. simplex* FAD-A1a and FAD-A1b were submitted to NCBI nr database under accession numbers OQ201605 and OQ201606, respectively. The complete protein-coding sequences of eight functionally characterized blattodean FADs were deposited to GenBank under accession numbers OP575963–OP575970. Other FADs discovered in in-house transcriptome assemblies are accessible under accession numbers OQ266404–OQ266432.

## References

[msad087-B1] Aboshi T , Shimizu N, Nakajima Y, Honda Y, Kuwahara Y, Amano H, Mori N. 2013. Biosynthesis of linoleic acid in *Tyrophagus* mites (Acarina: Acaridae). Insect Biochem Mol Biol. 43:991–996. 23973745 10.1016/j.ibmb.2013.08.002

[msad087-B2] Ando T , Inomata S-i, Yamamoto M. 2004. Lepidopteran sex pheromones. In: Schulz S, editors. The chemistry of pheromones and other semiochemicals I. Berlin, Heidelberg: Springer Berlin Heidelberg. p. 51–96.

[msad087-B3] Bade ML . 1964. Biosynthesis of fatty acids in the roach *Eurycotis floridana*. J Insect Physiol. 10:333–341.

[msad087-B4] Bai Y , McCoy JG, Levin EJ, Sobrado P, Rajashankar KR, Fox BG, Zhou M. 2015. X-ray structure of a mammalian stearoyl-CoA desaturase. Nature. 524:252–256. 26098370 10.1038/nature14549 PMC4689147

[msad087-B5] Blomquist GJ , Dwyer LA, Chu AJ, Ryan RO, de Renobales M. 1982. Biosynthesis of linoleic acid in a termite, cockroach and cricket. Insect Biochem. 12:349–353.

[msad087-B6] Bolger AM , Lohse M, Usadel B. 2014. Trimmomatic: a flexible trimmer for Illumina sequence data. Bioinformatics. 30:2114–2120. 24695404 10.1093/bioinformatics/btu170 PMC4103590

[msad087-B7] Bordereau C , Pasteels JM. 2011. Pheromones and chemical ecology of dispersal and foraging in termites. In: Bignell DE Roisin Y and Lo N, editors. Biology of termites: a modern synthesis. Dordrecht: Springer. p. 279–320.

[msad087-B8] Breznak JA , Brill WJ. 1973. Nitrogen fixation in termites. Nature. 244:577–580. 4582514 10.1038/244577a0

[msad087-B9] Brune A . 2014. Symbiotic digestion of lignocellulose in termite guts. Nat Rev Microbiol. 12:168–180. 24487819 10.1038/nrmicro3182

[msad087-B10] Buček A , Matoušková P, Vogel H, Šebesta P, Jahn U, Weißflog J, Svatoš A, Pichová I. 2015. Evolution of moth sex pheromone composition by a single amino acid substitution in a fatty acid desaturase. Proc Natl Acad Sci U S A. 112:12586–12591. 26417103 10.1073/pnas.1514566112 PMC4611599

[msad087-B11] Buček A , Šobotník J, He S, Shi M, McMahon DP, Holmes EC, Roisin Y, Lo N, Bourguignon T. 2019. Evolution of termite symbiosis informed by transcriptome-based phylogenies. Curr Biol. 29:3728–3734.e4. 31630948 10.1016/j.cub.2019.08.076

[msad087-B12] Buček A , Vazdar M, Tupec M, Svatoš A, Pichová I. 2020. Desaturase specificity is controlled by the physicochemical properties of a single amino acid residue in the substrate binding tunnel. Comp Struct Biotechnol J. 18:1202–1209. 10.1016/j.csbj.2020.05.011 PMC7283083 32542106

[msad087-B13] Buček A , Vogel H, Matoušková P, Prchalová D, Žáček P, Vrkoslav V, Šebesta P, Svatoš A, Jahn U, Valterová I, et al 2013. The role of desaturases in the biosynthesis of marking pheromones in bumblebee males. Insect Biochem Mol Biol. 43:724–731. 23727612 10.1016/j.ibmb.2013.05.003

[msad087-B14] Cai Y , Yu XH, Chai J, Liu CJ, Shanklin J. 2020. A conserved evolutionary mechanism permits Δ9 desaturation of very-long-chain fatty acyl lipids. J Biol Chem. 295:11337–11345. 32527722 10.1074/jbc.RA120.014258 PMC7415989

[msad087-B15] Carter FL , Dinus LA, Smythe RV. 1972. Fatty acids of the eastern subterranean termite, *Reticulitermes flavipes* (Isoptera: Rhinotermitidae). Ann Entomol Soc Am. 65:655–658.

[msad087-B16] Cripps C , Blomquist GJ, de Renobales M. 1986. De novo biosynthesis of linoleic acid in insects. Biochim Biophys Acta-Lipids Lipid Metab. 876:572–580.

[msad087-B17] Dallerac R , Labeur C, Jallon JM, Knipple DC, Roelofs WL, Wicker-Thomas C. 2000. A delta 9 desaturase gene with a different substrate specificity is responsible for the cuticular diene hydrocarbon polymorphism in *Drosophila melanogaster*. Proc Natl Acad Sci U S A. 97:9449–9454. 10920187 10.1073/pnas.150243997 PMC16884

[msad087-B18] de Renobales M , Cripps C, Stanley-Samuelson DW, Jurenka RA, Blomquist GJ. 1987. Biosynthesis of linoleic acid in insects. Trends Biochem Sci. 12:364–366.

[msad087-B19] Ding B-J , Carraher C, Löfstedt C. 2016. Sequence variation determining stereochemistry of a Δ11 desaturase active in moth sex pheromone biosynthesis. Insect Biochem Mol Biol. 74:68–75. 27163509 10.1016/j.ibmb.2016.05.002

[msad087-B20] Ding B-J , Liénard MA, Wang H-L, Zhao C-H, Löfstedt C. 2011. Terminal fatty-acyl-CoA desaturase involved in sex pheromone biosynthesis in the winter moth (*Operophtera brumata*). Insect Biochem Mol Biol. 41:715–722. 21651981 10.1016/j.ibmb.2011.05.003

[msad087-B21] Dunkelblum E , Tan SH, Silk PJ. 1985. Double-bond location in monounsaturated fatty acids by dimethyl disulfide derivatization and mass spectrometry: application to analysis of fatty acids in pheromone glands of four lepidoptera. J Chem Ecol. 11:265–277. 24309959 10.1007/BF01411414

[msad087-B22] Edgar RC . 2004. MUSCLE: multiple sequence alignment with high accuracy and high throughput. Nucleic Acids Res. 32:1792–1797. 15034147 10.1093/nar/gkh340 PMC390337

[msad087-B23] Esenther GR , Allen TC, Casida JE, Shenefelt RD. 1961. Termite attractant from fungus-infected wood. Science. 134:50. 10.1126/science.134.3471.50 17834302

[msad087-B24] Evangelista DA , Wipfler B, Béthoux O, Donath A, Fujita M, Kohli Manpreet K, Legendre F, Liu S, Machida R, Misof B, et al 2019. An integrative phylogenomic approach illuminates the evolutionary history of cockroaches and termites (Blattodea). Proceedings of the Royal Society B: Biological Sciences. 286:20182076. 10.1098/rspb.2018.2076 PMC6364590 30963947

[msad087-B25] Fang S , Ting CT, Lee CR, Chu KH, Wang CC, Tsaur SC. 2009. Molecular evolution and functional diversification of fatty acid desaturases after recurrent gene duplication in *Drosophila*. Mol Biol Evol. 26:1447–1456. 19307313 10.1093/molbev/msp057 PMC2693736

[msad087-B26] Fay L , Richli U. 1991. Location of double bonds in polyunsaturated fatty acids by gas chromatography-mass spectrometry after 4,4-dimethyloxazoline derivatization. J Chromatogr A. 541:89–98.

[msad087-B27] Gouy M , Guindon S, Gascuel O. 2010. Seaview version 4: a multiplatform graphical user interface for sequence alignment and phylogenetic tree building. Mol Biol Evol. 27:221–224. 19854763 10.1093/molbev/msp259

[msad087-B28] Grabherr MG , Haas BJ, Yassour M, Levin JZ, Thompson DA, Amit I, Adiconis X, Fan L, Raychowdhury R, Zeng Q, et al 2011. Full-length transcriptome assembly from RNA-Seq data without a reference genome. Nat Biotechnol. 29:644–652. 21572440 10.1038/nbt.1883 PMC3571712

[msad087-B29] Grimaldi DA , Engel MS. 2005. Evolution of the insects. Cambridge: Cambridge University Press.

[msad087-B30] Groot AT , Dekker T, Heckel DG. 2016. The genetic basis of pheromone evolution in moths. Annu Rev Entomol. 61:99–117. 26565898 10.1146/annurev-ento-010715-023638

[msad087-B31] Haritos VS , Horne I, Damcevski K, Glover K, Gibb N, Okada S, Hamberg M. 2012. The convergent evolution of defensive polyacetylenic fatty acid biosynthesis genes in soldier beetles. Nat Commun. 3:1150. 23093187 10.1038/ncomms2147

[msad087-B32] Harrison MC , Jongepier E, Robertson HM, Arning N, Bitard-Feildel T, Chao H, Childers CP, Dinh H, Doddapaneni H, Dugan S, et al 2018. Hemimetabolous genomes reveal molecular basis of termite eusociality. Nat Ecol Evol. 2:557–566. 29403074 10.1038/s41559-017-0459-1 PMC6482461

[msad087-B33] Helmkampf M , Cash E, Gadau J. 2015. Evolution of the insect desaturase gene family with an emphasis on social Hymenoptera. Mol Biol Evol. 32:456–471. 25425561 10.1093/molbev/msu315 PMC4298175

[msad087-B34] Hoang DT , Chernomor O, von Haeseler A, Minh BQ, Vinh LS. 2018. UFBoot2: improving the ultrafast bootstrap approximation. Mol Biol Evol. 35:518–522. 29077904 10.1093/molbev/msx281 PMC5850222

[msad087-B35] Holz C , Hesse O, Bolotina N, Stahl U, Lang C. 2002. A micro-scale process for high-throughput expression of cDNAs in the yeast Saccharomyces cerevisiae. Protein Expr Purif. 25:372–378. 12182816 10.1016/s1046-5928(02)00029-3

[msad087-B36] Jirošová A , Sillam-Dussès D, Kyjaková P, Kalinová B, Dolejšová K, Jančařík A, Majer P, Cristaldo PF, Hanus R. 2016. Smells like home: chemically mediated co-habitation of two termite species in a single nest. J Chem Ecol. 42:1070–1081. 27639394 10.1007/s10886-016-0756-1

[msad087-B37] Jouquet P , Traoré S, Choosai C, Hartmann C, Bignell D. 2011. Influence of termites on ecosystem functioning. Ecosystem services provided by termites. Eur J Soil Biol. 47:215–222.

[msad087-B38] Jumper J , Evans R, Pritzel A, Green T, Figurnov M, Ronneberger O, Tunyasuvunakool K, Bates R, Žídek A, Potapenko A, et al 2021. Highly accurate protein structure prediction with AlphaFold. Nature. 596:583–589. 34265844 10.1038/s41586-021-03819-2 PMC8371605

[msad087-B39] Jurenka R , Blomquist GJ, Schal C, Tittiger C. 2017. Biochemistry and molecular biology of pheromone production. In: Gilbert LI, editors. Comprehensive molecular insect science. Chapel Hill (NC): University of North Carolina. p. 705–751.

[msad087-B40] Knipple DC , Rosenfield CL, Nielsen R, You KM, Jeong SE. 2002. Evolution of the integral membrane desaturase gene family in moths and flies. Genetics. 162:1737–1752. 12524345 10.1093/genetics/162.4.1737 PMC1462379

[msad087-B41] Koubová J , Pangrácová M, Jankásek M, Lukšan O, Jehlík T, Brabcová J, Jedlička P, Křivánek J, Čapková Frydrychová R, Hanus R. 2021. Long-lived termite kings and queens activate telomerase in somatic organs. Proc R Soc B. 288:20210511. 10.1098/rspb.2021.0511 PMC8059557 33878922

[msad087-B42] Krishna K , Grimaldi DA, Krishna V, Engel MS. 2013. Treatise on the Isoptera of the world. Vol. 1. Introduction. Bull Amer Mus Nat Hist. 377:1–200.

[msad087-B43] Kyjaková P , Roy V, Jirošová A, Krasulová J, Dolejšová K, Křivánek J, Hadravová R, Rybáček J, Pohl R, Roisin Y, et al 2017. Chemical systematics of Neotropical termite genera with symmetrically snapping soldiers (Termitidae: Termitinae). Zool J Linn Soc. 180:66–81.

[msad087-B44] Langmead B , Salzberg SL. 2012. Fast gapped-read alignment with Bowtie 2. Nat Methods. 9:357–359. 22388286 10.1038/nmeth.1923 PMC3322381

[msad087-B45] Lassance JM , Groot AT, Lienard MA, Antony B, Borgwardt C, Andersson F, Hedenström E, Heckel DG, Löfstedt C. 2010. Allelic variation in a fatty-acyl reductase gene causes divergence in moth sex pheromones. Nature. 466:486–489. 20592730 10.1038/nature09058

[msad087-B46] Legendre A , Miao X-X, Da Lage JL, Wicker-Thomas C. 2008. Evolution of a desaturase involved in female pheromonal cuticular hydrocarbon biosynthesis and courtship behavior in *Drosophila*. Insect Biochem Mol Biol. 38:244–255. 18207084 10.1016/j.ibmb.2007.11.005

[msad087-B47] Lienard MA , Hagström AK, Lassance JM, Löfstedt C. 2010. Evolution of multicomponent pheromone signals in small ermine moths involves a single fatty-acyl reductase gene. Proc Natl Acad Sci U S A. 107:10955–10960. 20534481 10.1073/pnas.1000823107 PMC2890718

[msad087-B48] López Alonso D , García-Maroto F, Rodríguez-Ruiz J, Garrido JA, Vilches MA. 2003. Evolution of the membrane-bound fatty acid desaturases. Biochem Syst Ecol. 31:1111–1124.

[msad087-B49] Louloudes SJ , Kaplanis JN, Robbins WE, Monroe RE. 1961. Lipogenesis from C14-acetate by the American cockroach. Ann Entomol Soc Am. 54:99–103.

[msad087-B50] Malcicka M , Visser B, Ellers J. 2018. An evolutionary perspective on linoleic acid synthesis in animals. Evol Biol. 45:15–26. 29497218 10.1007/s11692-017-9436-5 PMC5816129

[msad087-B51] Matoušková P , Pichová I, Svatoš A. 2007. Functional characterization of a desaturase from the tobacco hornworm moth (*Manduca sexta*) with bifunctional *Z*11- and 10,12-desaturase activity. Insect Biochem Mol Biol. 37:601–610. 17517337 10.1016/j.ibmb.2007.03.004

[msad087-B52] Matsumura F , Coppel HC, Tai A. 1968. Isolation and identification of termite trail following pheromone. Nature. 219:963–964. 5673020 10.1038/219963a0

[msad087-B53] Mauldin JK , Rich NM, Cook DW. 1978. Amino acid synthesis from 14C-acetate by normally and abnormally faunated termites, *Coptotermes formosanus*. Insect Biochem. 8:105–109.

[msad087-B54] Mauldin JK , Smythe RV, Baxter CC. 1972. Cellulose catabolism and lipid synthesis by the subterranean termite, *Coptotermes formosanus*. Insect Biochem. 2:209–217.

[msad087-B55] Meesapyodsuk D , Qiu X. 2014. Structure determinants for the substrate specificity of acyl-CoA Δ9 desaturases from a marine copepod. ACS Chem Biol. 9:922–934. 24475735 10.1021/cb400675d

[msad087-B56] Mirdita M , Schütze K, Moriwaki Y, Heo L, Ovchinnikov S, Steinegger M. 2022. Colabfold: making protein folding accessible to all. Nat Methods. 19:679–682. 35637307 10.1038/s41592-022-01488-1 PMC9184281

[msad087-B57] Moto K , Suzuki MG, Hull JJ, Kurata R, Takahashi S, Yamamoto M, Okano K, Imai K, Ando T, Matsumoto S. 2004. Involvement of a bifunctional fatty-acyl desaturase in the biosynthesis of the silkmoth, *Bombyx mori*, sex pheromone. Proc Natl Acad Sci U S A. 101:8631–8636. 15173596 10.1073/pnas.0402056101 PMC423246

[msad087-B58] Nagao K , Murakami A, Umeda M. 2019. Structure and function of Δ9-fatty acid desaturase. Chem Pharm Bull. 67:327–332. 10.1248/cpb.c18-01001 30930436

[msad087-B59] Nguyen LT , Schmidt HA, von Haeseler A, Minh BQ. 2015. IQ-TREE: a fast and effective stochastic algorithm for estimating maximum-likelihood phylogenies. Mol Biol Evol. 32:268–274. 25371430 10.1093/molbev/msu300 PMC4271533

[msad087-B60] Odelson DA , Breznak JA. 1983. Volatile fatty acid production by the hindgut microbiota of xylophagous termites. Appl Environ Microbiol. 45:1602–1613. 16346296 10.1128/aem.45.5.1602-1613.1983 PMC242507

[msad087-B61] Pelley JW . 2012. 10—fatty acid and triglyceride metabolism. In: Pelley JW, editors. Elsevier’s integrated review biochemistry. 2nd ed. Philadelphia: W.B. Saunders. p. 81–88.

[msad087-B62] Pettersen EF , Goddard TD, Huang CC, Meng EC, Couch GS, Croll TI, Morris JH, Ferrin TE. 2021. UCSF Chimerax: structure visualization for researchers, educators, and developers. Protein Sci. 30:70–82. 32881101 10.1002/pro.3943 PMC7737788

[msad087-B63] Quast C , Pruesse E, Yilmaz P, Gerken J, Schweer T, Yarza P, Peplies J, Glöckner FO. 2012. The SILVA ribosomal RNA gene database project: improved data processing and web-based tools. Nucleic Acids Res. 41:D590–D596. 23193283 10.1093/nar/gks1219 PMC3531112

[msad087-B64] Roelofs WL , Rooney AP. 2003. Molecular genetics and evolution of pheromone biosynthesis in Lepidoptera. Proc Natl Acad Sci U S A. 100:14599. 10.1073/pnas.1233767100a PMC170892 12876197

[msad087-B65] Rothstein M , Götz P. 1968. Biosynthesis of fatty acids in the free-living nematode, *Turbatrix aceti*. Arch Biochem Biophys. 126:131–140. 5671057 10.1016/0003-9861(68)90567-5

[msad087-B66] Schneiter R , Tatzer V, Gogg G, Leitner E, Kohlwein SD. 2000. Elo1p-dependent carboxy-terminal elongation of C14:1Δ^9^ to C16:1Δ^11^ fatty acids in *Saccharomyces cerevisiae*. J Bacteriol. 182:3655–3660. 10850979 10.1128/jb.182.13.3655-3660.2000 PMC94535

[msad087-B67] Semmelmann F , Kabeya N, Malcicka M, Bruckmann A, Broschwitz B, Straub K, Merkl R, Monroig O, Sterner R, Ruther J, et al 2019. Functional characterisation of two Δ12-desaturases demonstrates targeted production of linoleic acid as pheromone precursor in *Nasonia*. J Exp Biol. 222:jeb201038. 10.1242/jeb.201038 31019064

[msad087-B68] Serra M , Pina B, Abad JL, Camps F, Fabrias G. 2007. A multifunctional desaturase involved in the biosynthesis of the processionary moth sex pheromone. Proc Natl Acad Sci U S A. 104:16444–16449. 17921252 10.1073/pnas.0705385104 PMC2034215

[msad087-B69] Shanklin J , Cahoon EB. 1998. Desaturation and related modifications of fatty acids. Annu Rev Plant Physiol Plant Molec Biol. 49:611–641. 15012248 10.1146/annurev.arplant.49.1.611

[msad087-B70] Shen J , Wu G, Tsai A-L, Zhou M. 2020. Structure and mechanism of a unique diiron center in mammalian stearoyl-CoA desaturase. J Mol Biol. 432:5152–5161. 32470559 10.1016/j.jmb.2020.05.017 PMC7483794

[msad087-B71] Shi H , Chen H, Gu Z, Song Y, Zhang H, Chen W, Chen YQ. 2015. Molecular mechanism of substrate specificity for delta 6 desaturase from *Mortierella alpina* and *Micromonas pusilla*. J Lipid Res. 56:2309–2321. 26486975 10.1194/jlr.M062158 PMC4655987

[msad087-B72] Shimizu N , Naito M, Mori N, Kuwahara Y. 2014. De novo biosynthesis of linoleic acid and its conversion to the hydrocarbon (*Z*, *Z*)-6,9-heptadecadiene in the astigmatid mite, *Carpoglyphus lactis*: incorporation experiments with 13C-labeled glucose. Insect Biochem Mol Biol. 45:51–57. 24333472 10.1016/j.ibmb.2013.11.006

[msad087-B73] Šobotník J , Weyda F, Hanus R, Cvačka J, Nebesářová J. 2006. Fat body of *Prorhinotermes simplex* (Isoptera: Rhinotermitidae): ultrastructure, inter-caste differences and lipid composition. Micron. 37:648–656. 16632370 10.1016/j.micron.2006.01.012

[msad087-B74] Song L , Florea L. 2015. Rcorrector: efficient and accurate error correction for Illumina RNA-seq reads. GigaScience. 4:48. 26500767 10.1186/s13742-015-0089-y PMC4615873

[msad087-B75] Sperling P , Ternes P, Zank TK, Heinz E. 2003. The evolution of desaturases. Prostaglandins Leukot Essent Fatty Acids. 68:73–95. 12538072 10.1016/s0952-3278(02)00258-2

[msad087-B76] Stanley-Samuelson DW , Jurenka RA, Cripps C, Blomquist GJ, de Renobales M. 1988. Fatty acids in insects: composition, metabolism, and biological significance. Arch Insect Biochem Physiol. 9:1–33.

[msad087-B77] Symonds MRE , Elgar MA. 2008. The evolution of pheromone diversity. Trends Ecol Evol. 23:220–228. 18308422 10.1016/j.tree.2007.11.009

[msad087-B78] Terrapon N , Li C, Robertson HM, Ji L, Meng X, Booth W, Chen Z, Childers CP, Glastad KM, Gokhale K, et al 2014. Molecular traces of alternative social organization in a termite genome. Nat Commun. 5:3636. 24845553 10.1038/ncomms4636

[msad087-B79] Tillman JA , Seybold SJ, Jurenka RA, Blomquist GJ. 1999. Insect pheromones—an overview of biosynthesis and endocrine regulation. Insect Biochem Mol Biol. 29:481–514. 10406089 10.1016/s0965-1748(99)00016-8

[msad087-B80] Tokoro M , Takahashi M, Yamaoka R. 1992. Identification of trail pheromone precursors from subterranean termite, *Coptotermes formosanus* Shiraki (Isoptera: Rhinotermitidae). J Chem Ecol. 18:517–526. 24254954 10.1007/BF00994249

[msad087-B81] Tokoro M , Yamaoka R, Hayashiya K, Takahashi M, Nishimoto K. 1990. Evidence for trail-pheromone precursor in termite *Reticulitermes* speratus (Kolbe) (Rhinotermitidae: Isoptera). J Chem Ecol. 16:2549–2557. 24264219 10.1007/BF01017477

[msad087-B82] Tuma J , Eggleton P, Fayle TM. 2020. Ant-termite interactions: an important but under-explored ecological linkage. Biol Rev. 95:555–572. 31876057 10.1111/brv.12577

[msad087-B83] Tupec M , Buček A, Janoušek V, Vogel H, Prchalová D, Kindl J, Pavlíčková T, Wenzelová P, Jahn U, Valterová I, et al 2019. Expansion of the fatty acyl reductase gene family shaped pheromone communication in Hymenoptera. eLife. 8:e39231. 10.7554/eLife.39231 PMC6361591 30714899

[msad087-B84] Tupec M , Buček A, Valterová I, Pichová I. 2017. Biotechnological potential of insect fatty acid-modifying enzymes. Z Naturforsch C. 72:387–403. 28742527 10.1515/znc-2017-0031

[msad087-B85] Vanhercke T , Shrestha P, Green AG, Singh SP. 2011. Mechanistic and structural insights into the regioselectivity of an acyl-CoA fatty acid desaturase via directed molecular evolution. J Biol Chem. 286:12860–12869. 21300802 10.1074/jbc.M110.191098 PMC3075633

[msad087-B86] Wang H , Klein MG, Zou H, Lane W, Snell G, Levin I, Li K, Sang BC. 2015. Crystal structure of human stearoyl-coenzyme A desaturase in complex with substrate. Nat Struct Mol Biol. 22:581–585. 26098317 10.1038/nsmb.3049

[msad087-B87] Wang H-L , Liénard MA, Zhao C-H, Wang C-Z, Löfstedt C. 2010. Neofunctionalization in an ancestral insect desaturase lineage led to rare Δ6 pheromone signals in the Chinese tussah silkworm. Insect Biochem Mol Biol. 40:742–751. 20691782 10.1016/j.ibmb.2010.07.009

[msad087-B88] Weinert J , Blomquist GJ, Borgeson CE. 1993. De novo biosynthesis of linoleic acid in two non-insect invertebrates: the land slug and the garden snail. Experientia. 49:919–921.

[msad087-B89] Wilding M , Nachtschatt M, Speight R, Scott C. 2017. An improved and general streamlined phylogenetic protocol applied to the fatty acid desaturase family. Mol Phylogenet Evol. 115:50–57. 28739372 10.1016/j.ympev.2017.07.012

[msad087-B90] Wilson EO . 1992. Social insects as dominant organisms. In: Billen J, editors. Biology and evolution of social insects. Leuven: Leuven University Press. p. 1–7.

[msad087-B91] Zhou XR , Horne I, Damcevski K, Haritos V, Green A, Singh S. 2008. Isolation and functional characterization of two independently-evolved fatty acid Delta12-desaturase genes from insects. Insect Mol Biol. 17:667–676. 19133076 10.1111/j.1365-2583.2008.00841.x

