# Supplementary material for: Evolution of Linoleic Acid Biosynthesis Paved the Way for Ecological Success of Termites
Source: Mol Biol Evol. 2023 Apr 12;40(4):msad087. doi: 10.1093/molbev/msad087 (PMC10139705; doi:10.1093/molbev/msad087)
Supplement: msad087_Supplementary_Data [file msad087_supplementary_data.zip › SUPPLEMENTARY MATERIAL ONLINE.pdf]

```
Psim-A1a              1 MAPN----------ITSSPTGVL-------FEDDIVETVAVSSVEPKN--HDSKPPQKYSRQIVWRNVILFVYLHLAAVY
Pame-A1a              1 MAPN----------ITSSPTGVL-------FEDDTIETVTLPTIETKT--DDSKPAEKYKRKIVWRNVILFVYLHMAALY
Znev-A1a              1 MAPN----------ITSSPTGVL-------FEDDIVETVAVPSIESKN---DAKPPEKYRRQIVWRNVILFAYLHLAAVY
Eneo-A1a              1 MAPN----------ITGSPTGVL-------FEDDTVETVTVPSVEPKN--HDSKPPENYRRQIVWRNVILFVYLHVAAVY
Tcas-ADK13055.1       1 MTPN-----------ASIPTGVL-------HEND--EEVSNATLPPEV----NKPDDR-KLQLVWRNIILFAYLHLAALY
Clug-AFJ66833.1       1 MAPN-----------ISAPTGVL-------HETD--EEVSVPQLQQRE---EKAATPKPPLKIVWRNVIIFVYLHTVSLY
Adom-Q9BH41           1 MAPN----------ITSAPTGVL-------FEGDTIGPAAKDQQAEVNAPEAKKPREPYRRQIVWRNVILFIYLHLAALY
Psim-A1b              1 MAPN----------VTSSPTEVL-------CTPE---TTLHSSNEIKD--GIEKPALKYRREIVWPNVVMHIYLNLAALY
Pame-A1b              1 MAPN----------ITSTPTGVL-------YEEDFAAAEKATSTETKE---GIKPKREYKKQIVWPNVIMHFLLHVGAVY
Znev-A1b              1 MAPN-----------SSSVTEDR-------GAGD---TAHAPQNERRG--AGAEPYPNYRTEVVWPNVVVHAYLNLAALY
Eneo-A1b              1 MAPN----------VTSSATEVL-------CTPE---TTLHSSSEIRA--GVEKPTLKYRREIVWPNVVMHIYLNVAALY
Tcas-B7SB92           1 MSAQ-----------------------------------TITTTETTQ---NAQKPQQYHWRMVWRNIILYIIMHLTGFY
Clug-AFJ66832.1       1 MAPN-----------ARDSTGVL-------SEED--AEITPEQIEEVH-----KTSKNRHLPLVWRDVIKFAVLHALGFY
Adom-B7SB91           1 MDLN----------EESAPSGVL-------FEEDVAEQEAKMANGGPK--KGKKLEEPYRLEIVWFNVLWFVLLHAGALY
Nvit-XP_001599836.1   1 MPPNEQIYEHILPEAVEDPLKTLDIPGFDGYDEQYKDSEFNMSAEEYKEKVLSKPFLQ-RTDIKWGNLLFMVVMHAVALY

                                                                    substrate acyl tunnel
Psim-A1a             62 GAYLMLTSAKIITTVFAVLLYQAGGLGITAGAHRLWAHRSYKAKWPLRLLLVIFNTLAFQNDVCEWARDHRVHHKFSETD
Pame-A1a             62 GAYLMLTSCKLITAIWAILLYQAGGLGITAGAHRLWSHRAYKAKWPLRLILVIFNTLAFQNHVYEWARDHRVHHKFSETD
Znev-A1a             61 GAYLMLTSAKIITSIWAILLYQAGGLGITAGAHRLWAHRAYKAKWPLRLILMLFNTLAFQNHVHEWARDHRVHHKFSETD
Eneo-A1a             62 GAYLMLTSAKIITTVWAILLYQAGGLGITAGAHRLWAHRAYKAKWPLRLILVIFNTLAFQNDVCEWARDHRVHHKFSETD
Tcas-ADK13055.1      56 GIWIMFTSAKVATSLFGILLYQLGGFGITAGAHRLWAHRSYKAKWPLRLILTFCNTLAFEDSVIDWSRDHRVHHKFSETD
Clug-AFJ66833.1      58 GIYLIFSSAKIATTILAFFLYNLGGLGITAGAHRLWAHRSYKAKWPLRLLLTTFNTLAFQDAVVDWARDHRVHHKYSETD
Adom-Q9BH41          64 GAYLAFTSAKIATTIFAIILYQVSGVGITGGAHRLWAHRSYKAKWPLRVILMLCNTLAFQNHIYEWARDHRVHHKFSETD

Psim-A1b             59 GAYMMLTSAKLLTGLWAFILYEGGILGITAGAHRLWSHRAYRATWQLRLILMLLQTLAFQMSVHEWVRNHRVHHKNSDTD
Pame-A1b             61 GAYLMLTSAKILTGIWAFFLYEVGILGITAGAHRLWSHRSYKATWQMRLILMICQTVSFQTSVHEWARNHRVHHKHSDTD
Znev-A1b             58 GAYLMFTSARLPTAIWAFILYEAGILGITAGAHRLWSHRAYKATWPLRVFLVLLQTLAFQTSAHEWVRNHRVHHKFSDTD
Eneo-A1b             59 GTYLMLTSAKILTGIWAFILYEGGILGITAGAHRLWSHRAYKATWQLRLILMLLHTLAFQTSVHEWVRNHRVHHKNSDTD
Tcas-B7SB92          43 GLYLAMFYAQWKTVFYSWFLLVIALQGVTAGSHRLWAHKAYKARLPLRMLLCIFQTLSLQNHIYDWATYHRVHHKFVDTN
Clug-AFJ66832.1      56 GFYLMFTSARIWTSVLVFINYHLGILGITAGAHRLWAHKAYKARWPLKLFLAYIQTLAFQFDIIFWAKYHRVHHKYTETD
Adom-B7SB91          62 GVYLIFASAKIYTTLYGFLLCELSLLSITAGVHRLWAHRAYKAKWPLRLTLMVLNLLAYQNSIYEWARDHRVHHKFSETN
Nvit-XP_001599836.1  80 GFLTYPYLEKKMTFVWGWFLAIVANFGVASGVHRLWSHRAFKAKLPFKIISIICYLTSGQYSAIWWSRDHRLHHKFSETD
Fe binding site                                          1    1                               1   21

                             substrate acyl tunnel
Psim-A1a            142 ADPHNATRGFFFSHVGWLLVRKHPDVKEKGKTIDMSDLEADPFLRFQKKYYLLVM-PLLCFVLPTLVPVYLWGETWSNAW
Pame-A1a            142 ADPHNAKRGFFFSHVGWLLVRKHPDVKVKGKGIDMSDLDADPLIAFQKKHYLILM-PIICFILPTIIPVYFWGETWSNAW
Znev-A1a            141 ADPHDARRGFFFSHVGWLLVRKHPDVKEKGKGIDMSDLDTDPLLMFQKKYYLIMM-PILCFLIPTMVPVYLWGESWTNAW
Eneo-A1a            142 ADPHNATRGFFFSHVGWLLVRKHPDVKEKGKAIDMSDLEADPFLRFQKKYYLIVM-PILCFLVPTCVPVYLWGETWSNAW
Tcas-ADK13055.1     136 ADPHNAKRGFFFSHVGWLLCRKHPQVKEKGKQIDLSDLYADPILRFQKKYYTIVM-PLVSFVMPTVVPMYLWGETFKNA-
Clug-AFJ66833.1     138 ADPHNATRGFFFAHVGWLLCRKHPDVKAKGSGIDLSDLYSDPILRYQKKFYLLLM-PLVCFVVPTSIPMYLWNETFMNA-
Adom-Q9BH41         144 ADPHNATRGFFFSHVGWLLVRKHPDVKEKGKGIDMHDLEQDKIVMFQKKYYLILM-PIVCFLIPTTIPVYMWNETWSNAW

Psim-A1b            139 ADPHNVRRGFFFTHYGWMLVRKHPAVKEKGKKIDWSDLDADALLVFQKRYYIILM-PVLCFLIPSAVPVYLWGETWTNAY
Pame-A1b            141 GDPHNVNRGLFFSHAGWMMCRKHPEVKEKGKQIDLSDLDADPILMFQKKYYLILM-PFMCFFLPTWIPVYFWGETWHNAY
Znev-A1b            138 ADPHNVNRGFFFTHYGWMLTKKHPAVKENGKKVDSSDLDADPLLIFQKRYYMIIM-PILCFVIPTAVPVFVWNETWSNAY
Eneo-A1b            139 ADPHNVHRGFFFTHYGWMLVRKHPAVKEKGKLIDWSDLDADQLLLFQKRYYLILM-PILCFLIPSAVPVYLWGETWTNSY
Tcas-B7SB92         123 ADPHNSRRGFFFSHMGWLFIEPHKDVEDKYKSIDFSDLHADSVVMIQKKYYHTFFAPVIGFYLPAAIPWYFWGENFWTAF
Clug-AFJ66832.1     136 ADPHNAKRGFFYSHMGWTMVKKNPEFETRCNEIDLSDLYSDPIVNFQYKYYYQIL-FVVFLVVPTFIPMYFWNETFVNA-
Adom-B7SB91         142 ADPVNAKRGFFFSHVGWLLCRKHPEVRAKGGRIDLSDLERDPIVMFQKRHYYKLV-PFVSFVIPTLIPMYFWGETLSNSW
Nvit-XP_001599836.1 160 ADPVNAARGFWFSHIGWLCMKRHPEVLKKAKQLDLSDIANDPVIKFEEKHFQVLR-LMFTFIIPTLVPVYFWNESWYWAI

                              substrate acyl tunnel
Psim-A1a            221 FVVAMFRYTFTLNMTWLVNSAAH---FWGNRPYDKYINPAENLGVAVFALGEGWHNYHHVFPWDYKTAELGNY---RTNL
Pame-A1a            221 FVVAMFRYTFTLNASWLVNSAAH---MWGSRPYDKYINPSENLGVSMLALGEGWHNYHHVFPWDYKTAELGNY---STNL
Znev-A1a            220 FVVAMFRYTFTLNMTWLVNSAAH---MWGSQPYDKYINPAENLSVAVLALGEGWHNYHHVFPWDYKTAELGNY---RANL
Eneo-A1a            221 FVVAMFRYAFTLNMTWLVNSAAH---IWGTRPYDKYINPAENLGVAVFALGEGWHNYHHVFPWDYKTAELGNY---RANL
Tcas-ADK13055.1     214 FLVNLFRYCLTLNATWLVNSAAH---MWGDKPYDRFINPAENFVVSVLALGEGWHNYHHTFPWDYKTSELGKY---SVNF
Clug-AFJ66833.1     216 FCVNLFRYTFTLNATWLVNSAAH---LFGDKPYDRFINPAENVSVAVLAMGEGWHNYHHTFPWDYKTSELGKY---STNV
Adom-Q9BH41         223 FVATLFRYTFTLNMTWLVNSAAH---MWGSQPYDKYINPAENLGVALGAMGEGWHNYHHVFPWDYKAAELGNY---RANF

Psim-A1b            218 HIAAVLRQVVTLHMTLITNSITHWPDTWRKRPYDKNIFPSENFMVSLLTLGEGWHNFHHVFPWDYKTAEIGGN---RINP
Pame-A1b            220 FVAAIFRHVFTLNMTLMVNSITH--NTWGNRPYDKNINPAENAIVSLMTLGEGWHNYHHVFPWDYKTAELGVL---RINM
Znev-A1b            217 HIAAVMRQVITLHMTLITNSVTHWPDTWRKRPYDKRICPSENFFVSLLTLGEGWHNFHHVFPWDYKTSEIGYN---RINP
Eneo-A1b            218 HIAAVLRQVVTLHMTLITNSITHWPDTWRKRPYDKNICPSENFMVSLLTLGEGWHNFHHVFPWDYKTAEIGGN---RINP
Tcas-B7SB92         203 FVATMLRYCACTNITFLVNSWAH---IYGSRPYDKNIYPTESATIAVLTGGEGWHNYHHTFPWDYKTGEFGKY---RSNL
Clug-AFJ66832.1     214 FCLNLSRYLLSLHCTWLVNSAAH---LYGNKPYDKSLYSSENFWVTILVNGEGWHNYHHAFPWDYKASELGIY---STNM
Adom-B7SB91         221 YVSTMFRYCLSLNLTWLVNSAAH---MWGNKPYDKNINPVENLAVAIGSLGEGWHNFHHVFPWDYKTSELGNY---SLNF
Nvit-XP_001599836.1 239 LSQCFMRYVYSLNCTWGINSLAH---RFGGHPYDKNIEPSENFLMTLATGGEGWHNYHHAFPADYKASELGFNYITNVNL
Fe binding site                              2   2                                 2  12

Psim-A1a            295 TTAFIDFFSRIGWAYDLKTVPLSMVKRRVERTGDGSHE------------------VWGWGDKDMSQEDMDEAQVINKKVT
Pame-A1a            295 TTAFIDFFSRIGWAYDLKTVPMSMVKQRVQRTGDGSHD------------------VWGWGDKDMSQEDMDEAQVINKKLK
Znev-A1a            294 TTAFIDFFSRIGWAYDLKTVPMSMVKRRVERTGDGSHE------------------VWGWGDKDMSQDDMDQAQVINNKSK
Eneo-A1a            295 TTAFIDFFSRIGWAYDLKTVPLSMVKRRVERTGDGSHE------------------VWGWGDKDMSQEDMDEAQVINKKVK
Tcas-ADK13055.1     288 STAFIDFFAKIGWAYDLKTVSSEMIKKRVTRTGDGTHE------------------IWGWGDKDQSQEDYQDAIITHRKSA
Clug-AFJ66833.1     290 TTAFIDLMAKIGWAYELKTVPMEMIKKRVERTGDGTHE------------------LWGWGDKDQPIEERKDALITHVKSF
Adom-Q9BH41         297 TTAFIDFFARIGWAYDLKTVPVSMIQRRVERTGDGSHE------------------VWGWGDKDMPQEDIDGAVIEKRKTQ
Psim-A1b            295 TTRFIDFCARIGWAYDLKTVPMSVVKRRIERTGDGTHE------------------IWGWGDKDMSTRDKSLVEVVNQEAK
Pame-A1b            295 TTLFIDLCAKIGWAYDLKTVPMDMVKRRVERTGDGTHE------------------IWGWGDKDMTEKEREIAQIINKKD-
Znev-A1b            294 TTHFIDFCARIGLAYDLKTVPTSMVKRRVQRTGDGTHE------------------LWGWGDADMSEAEKKMVQVVNEKAT
Eneo-A1b            295 TTHFIDFCARIGWAYDLKTVPMSVVKKRVERTGDGTHE------------------IWGWGDKDMTTSEKNLVYVINKKVM
Tcas-B7SB92         277 TTGFLDFMAAIGWAYDLKTVSEEMIMKRVLRTGDGTRKFDKIDKILNVDDDHHHEDMLWGWGDSDMAKEEMNYVKIHNRKED
Clug-AFJ66832.1     288 TAFFIDAMAKLGLAYDLKSVAPEVVKRRVERTGDGSHP------------------IWGWGDKDQSEEDKRSALISH-KDT
Adom-B7SB91         295 TNAFIDLAVLLGLAYDLKTVPVSMIKTRVGRTGDGSHD------------------VWGWGDKDLPKELADQTMIENRKTE
Nvit-XP_001599836.1 316 TTCLIDLAAKIGWAYDLKEPSEKLLKAVIKNRGDGSHP-------------------------ISHETVKT--------E
```

Supplementary Figure 1. Alignment of FAD-A1a and FAD-A1b amino acid sequences from four blattodean species compared to previously functionally characterized Δ9 and Δ12 FADs from other insects. Fully conserved residues are highlighted in dark shading, residues involved in substrate binding are bold-faced. Transmembrane domains are highlighted by blue boxes, Fe ion-binding sites are in red with the corresponding ion identifier underneath. Residues forming the internal region of the substrate tunnel in *P. simplex* enzymes are marked in green and interconnected by green horizontal bars and correspond to residues depicted in fig. 2B. Sequences were aligned using MUSCLE v5 algorithm. The alignment region used for phylogenetic analysis is marked with a black line. This figure is a full version of the partial alignment shown in fig. 2A of the main text.

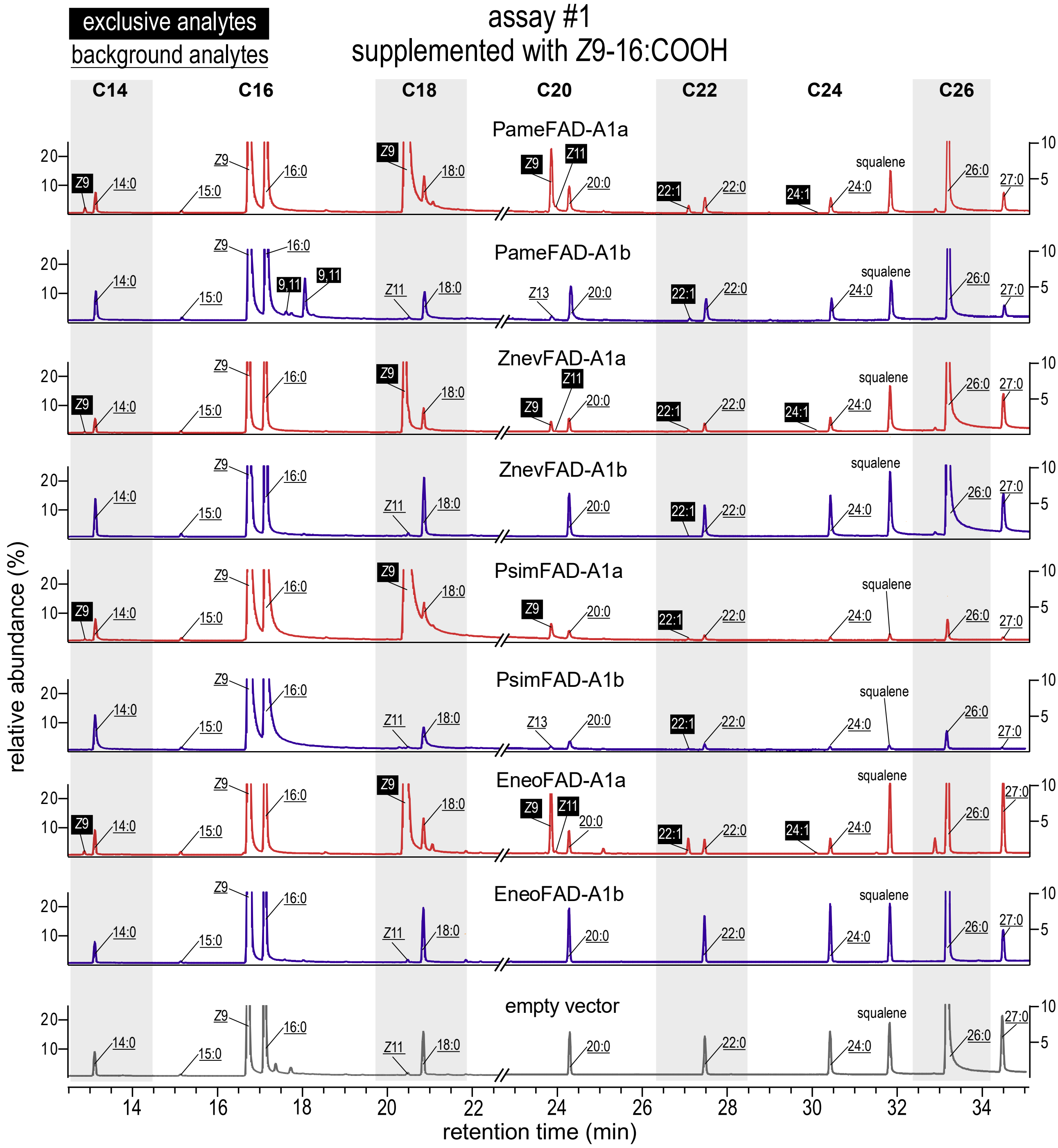

Supplementary Figure 2. GC chromatograms from functional assays with FAD-A1a and FAD-A1b of one cockroach (*P. americana*) and three termite species from three different families (related to fig. 3 left). The yeast were transformed with constructs containing full coding sequences of the studied FADs, and cultured for 4 days in media supplemented with *Z*9-16:COOH. Prior to GC analyses, the extracts were transesterified to convert fatty acids into methyl esters (FAMEs). Identifications of individual FAMEs is supported by supplementary fig. 4.

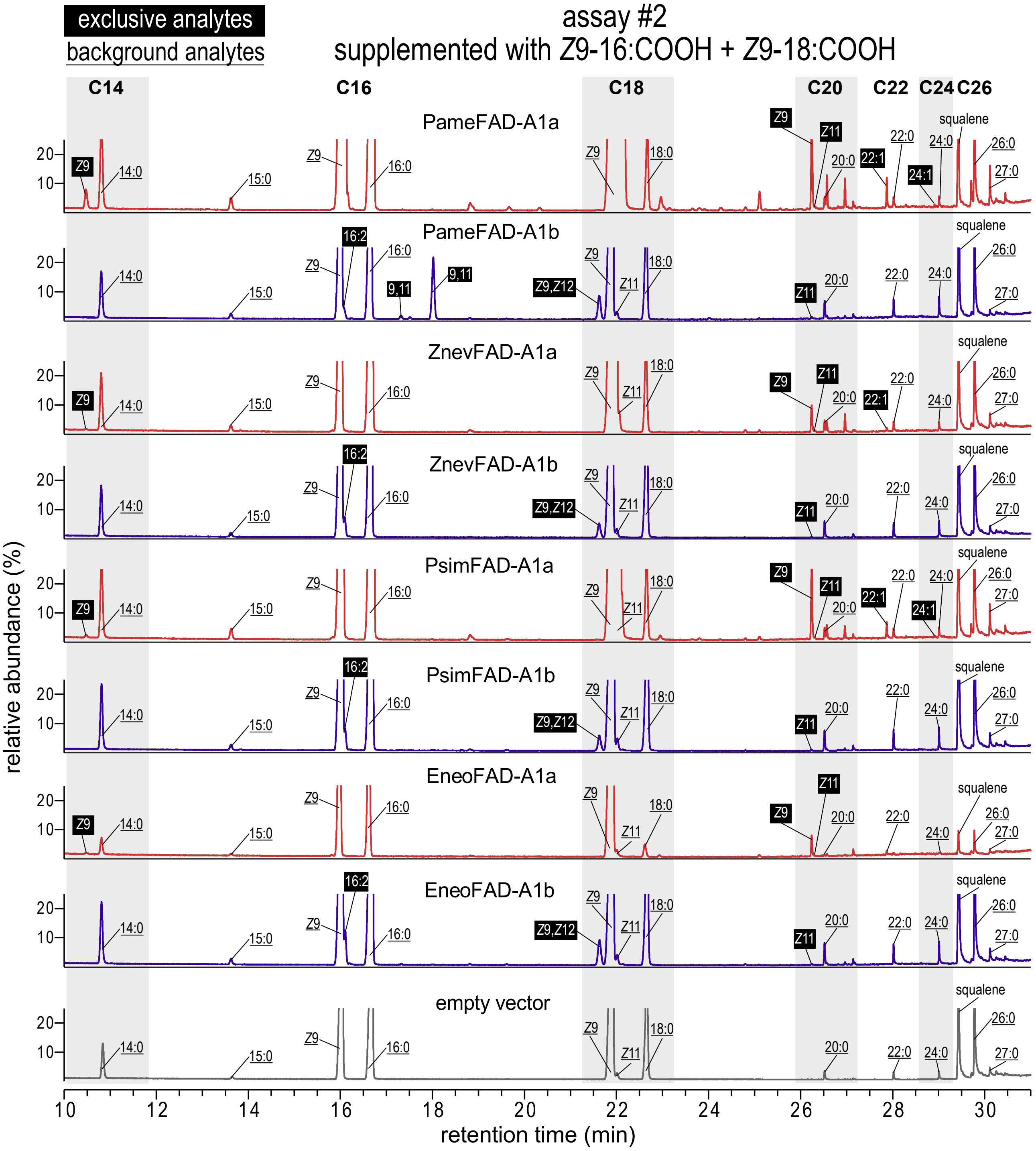

Supplementary Figure 3. GC chromatograms from functional assays with FAD-A1a and FAD-A1b of one cockroach (*P. americana*) and three termite species from three different families (related to fig. 3 right). The yeast were transformed with constructs containing full coding sequences of the studied FADs, and cultured for 4 days in media supplemented with *Z*9-16:COOH and *Z*9-18:COOH. Prior to GC analyses, the extracted fatty acyls were transesterified into methyl esters (FAMEs). Identifications of individual FAMEs is supported by supplementary fig. 4.

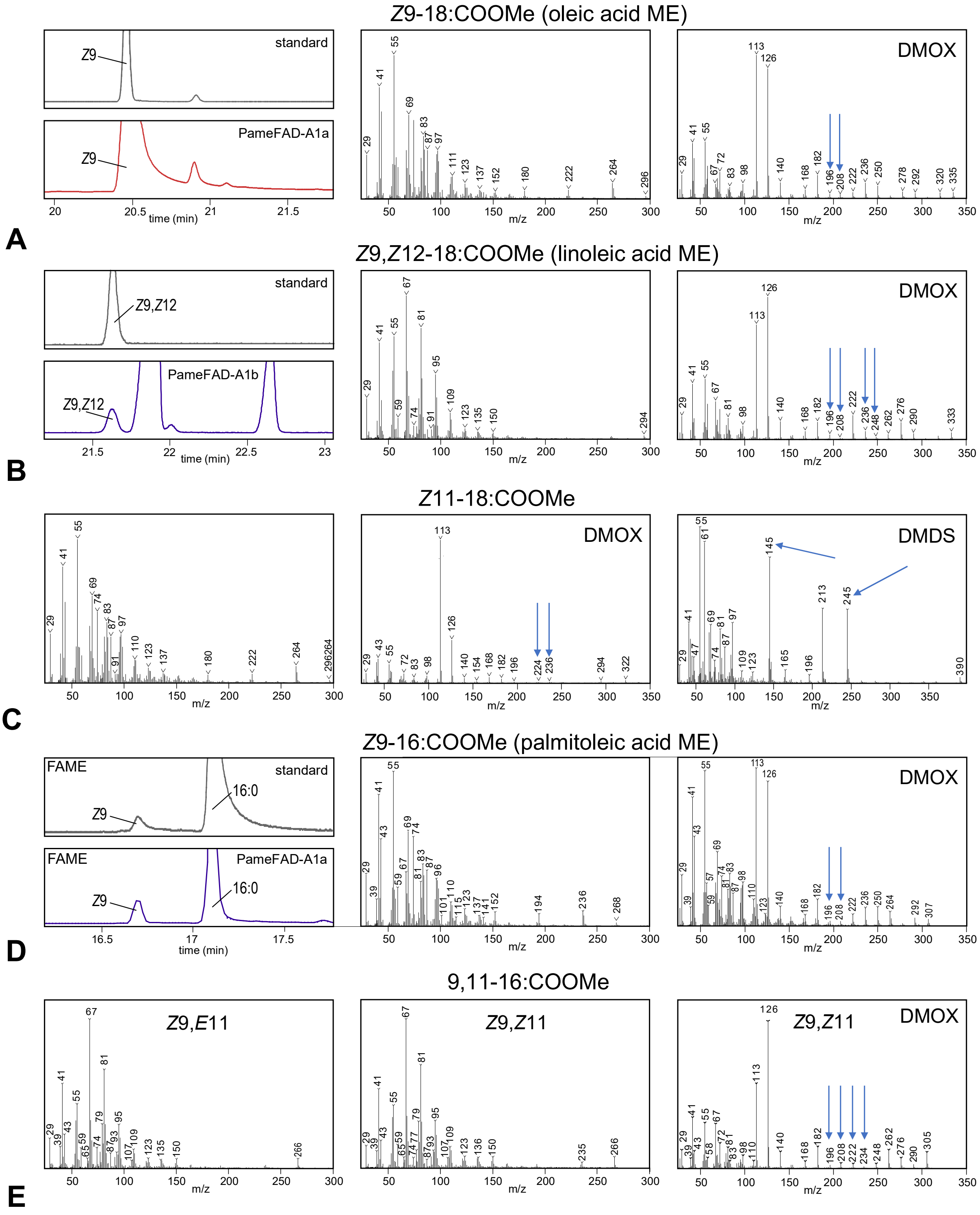

Supplementary Figure 4. Identification of C16 and C18 enzymatic products of FAD-A1a and FAD-A1b in yeast (related to fig. 3). **A.** FAD-A1a produces *Z*9-18:COOH (oleic acid) from 18:COOH (stearic acid). **B.** FAD-A1b produces *Z*9,*Z*12-18:COOH (linoleic acid) from oleic acid. **C.** *Z*11-18:COOMe occurs in all incubations (including control) containing *Z*9-16:COOH, presumably via the action of fatty acid elongases. **D.** FAD-A1a produces *Z*9-16:COOH from 16:COOH (palmitic acid). **E.** Extracts from cultures of *P. americana* FAD-A1b supplemented with *Z*9-16:COOH contain two stereoisomers of 9,11-unsaturated 16:COOMe. According to their elution order, these were tentatively identified as *Z*9,*E*11-16:COOMe and *Z*9,*Z*11-16:COOMe. The lipids extracted from yeast were transesterified and the resulting fatty acyl methyl esters (FAMEs) further derivatized using DMOX and/or DMDS techniques. Retention characteristics and mass spectra of detected FAMEs and DMOX- or DMDS-derivatized FAMEs were compared with those of synthetic standards, previously reported data (Buser et al. 1983; Fay and Richli 1991), and/or expected MS fragmentation patterns. Depicted chromatograms and mass spectra report the situation in *P. americana* (i.e., in *Pame*FAD-A1a and *Pame*FAD-A1b). Identical approach was used for the identification of observed C14, C20, C22, C24 and C26 unsaturated FAME homologs (data not shown).

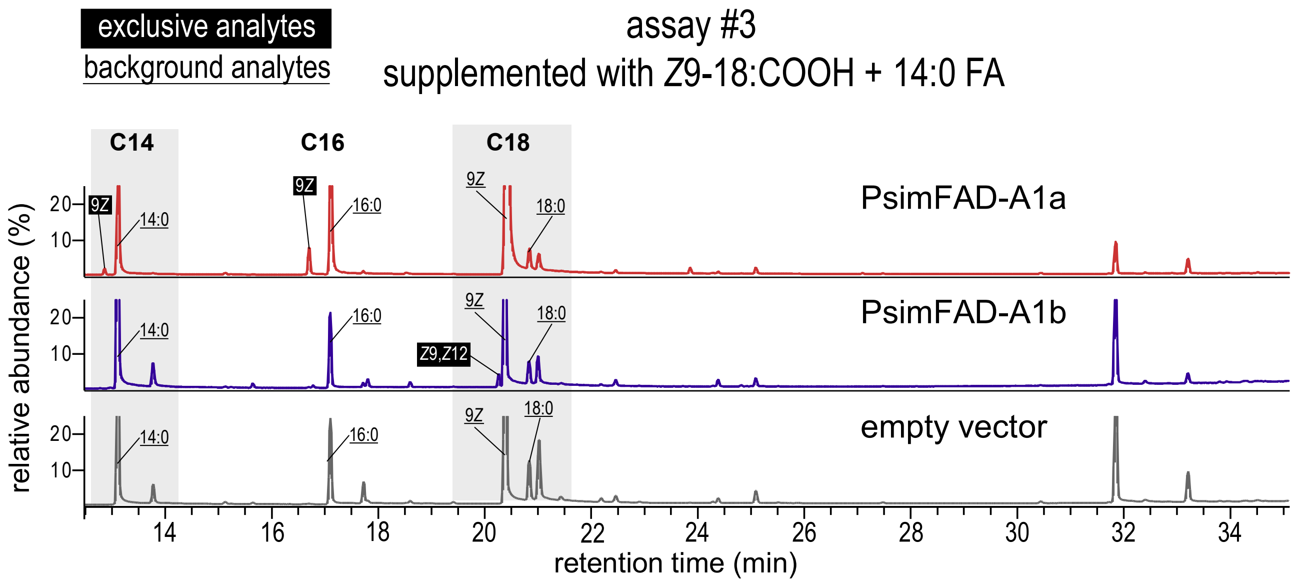

Supplementary Figure 5. Substrate specificity of FAD-A1a and FAD-A1b in the termite *P. simplex*. FAD-A1a produces *Z*9-16:COOH from 16:COOH and *Z*9-14:COOH from 14:COOH. Empty vector serves as a negative control. The lipids extracted from yeast were transesterified and the resulting fatty acyl methyl esters (FAMEs) further derivatized using DMOX and/or DMDS techniques. Retention characteristics and mass spectra of detected FAMEs and DMOX- or DMDS-derivatized FAMEs were compared with those of synthetic standards, previously reported data (Buser et al. 1983; Fay and Richli 1991), and/or expected MS fragmentation patterns.

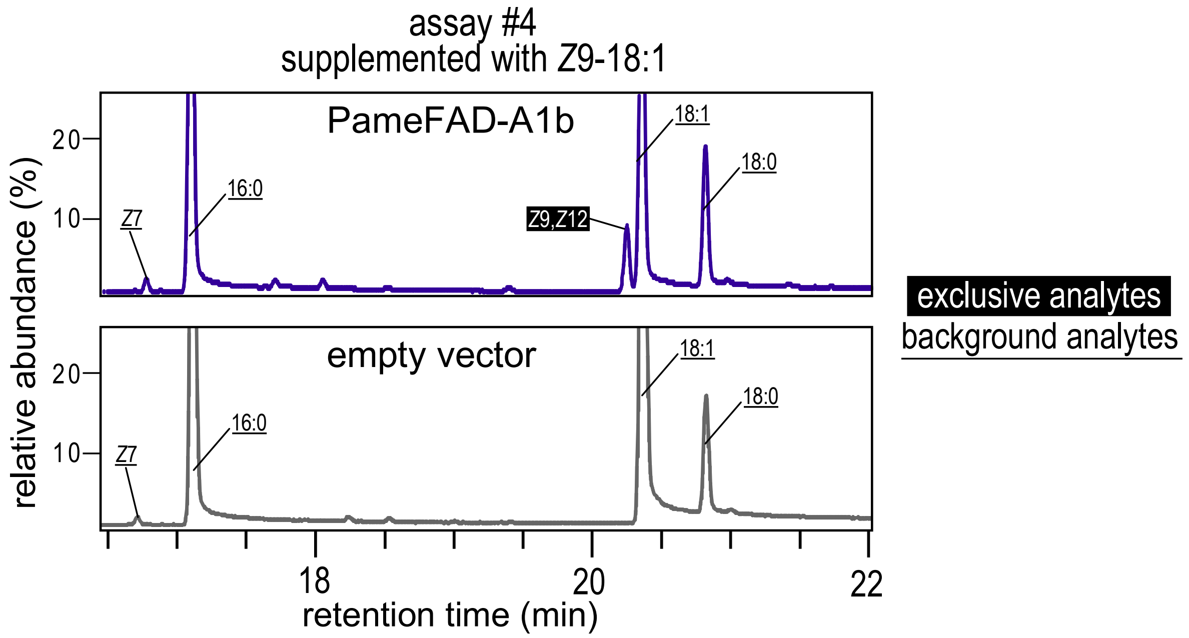

Supplementary Figure 6. Identification of C16 and C18 enzymatic products of FAD-A1b from the cockroach *P. americana* when yeast culture was supplemented with *Z*9-18:COOH only, i.e. without any *Z*9-16:COOH. Empty vector serves as a negative control. The enzyme produces *Z*9,*Z*12-18:COOH (linoleic acid) but not the two C16:2 isomers observed when *Z*9-16:COOH is supplemented (fig. 3), suggesting that these do not arise from *Z*9,*Z*12-18:COOH shortening, but rather from desaturase activity on *Z*9-16:COOH which is specific to *Pame*FAD-A1b. Both chromatograms also show *Z*7-16:COOH, likely originating from shortening of the supplemented *Z*9-18:COOH. The lipids extracted from yeast were transesterified and the resulting fatty acyl methyl esters (FAMEs) further derivatized using DMOX and/or DMDS techniques. Retention characteristics and mass spectra of detected FAMEs and DMOX- or DMDS-derivatized FAMEs were compared with those of synthetic standards, previously reported data (Buser et al. 1983; Fay and Richli 1991), and/or expected MS fragmentation patterns.

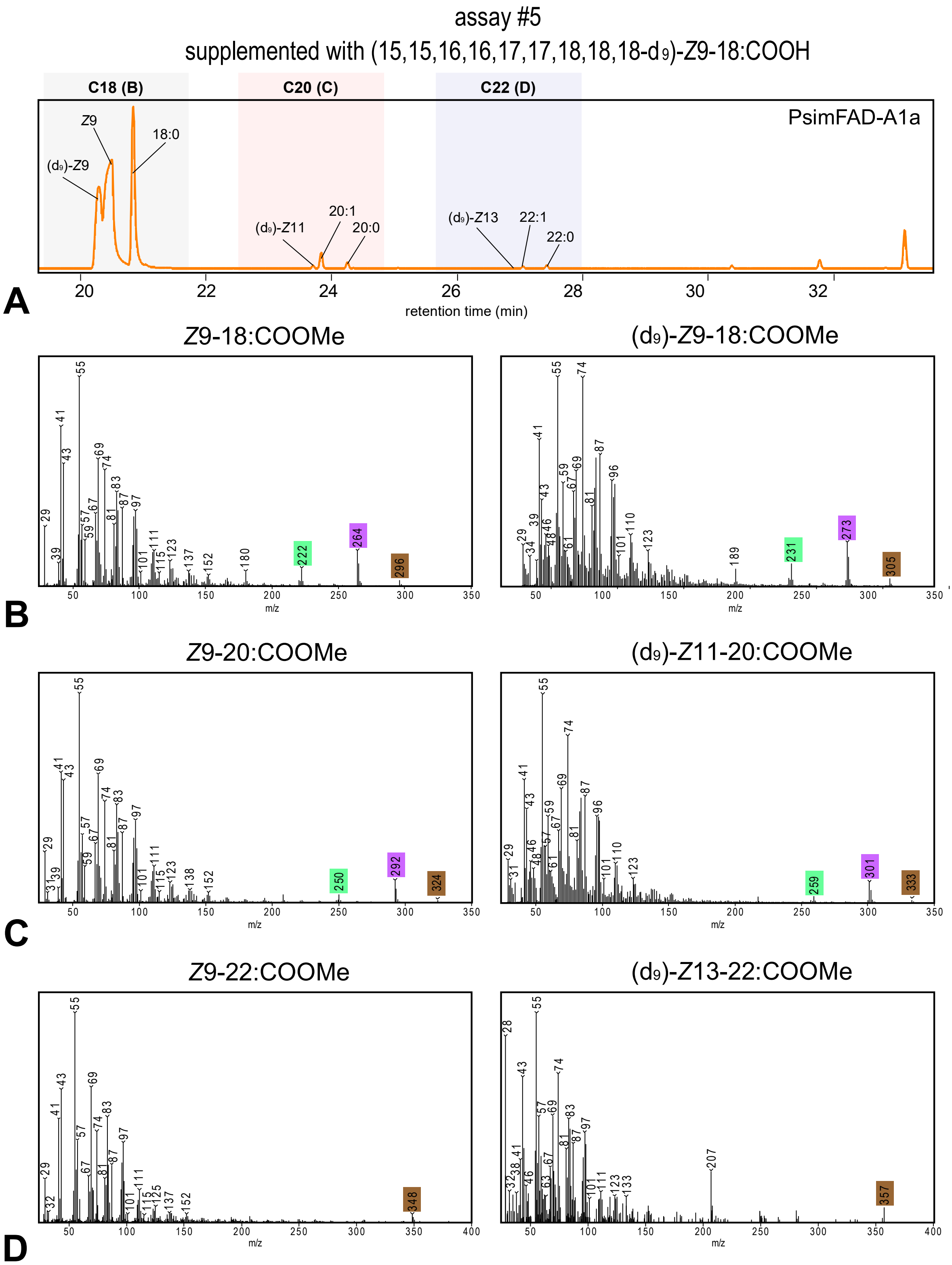

Supplementary Figure 7. Evidence of intrinsic FA elongation in yeast demonstrated using a culture of *Psim*FAD-A1a supplemented with (15,15,16,16,17,17,18,18,18-d_9)-oleic acid. **A.** The chromatogram shows methyl ester of oleic acid (*Z*9-18:COOMe) arising from native stearic acid, methyl ester of the supplemented (d_9)-OA, and of C20 and C22 monounsaturated FAs. The deuterated C20 and C22 FAMEs were identified as (d_9)-11-20:COOMe and (d_9)-13-22:COOMe, both having *Z* double bond configurations due to their origin via elongation of (d_9)-OA. **B.** Mass spectra of native (left) and deuterated *Z*9-18:COOMe (right). **C.** Mass spectra of native C20:1 FAME (left) and deuterated *Z*11-20:COOMe (right) elongation products. **D.** Mass spectra of native C22:1 FAME (left) and deuterated *Z*13-22:COOMe (right) elongation products.

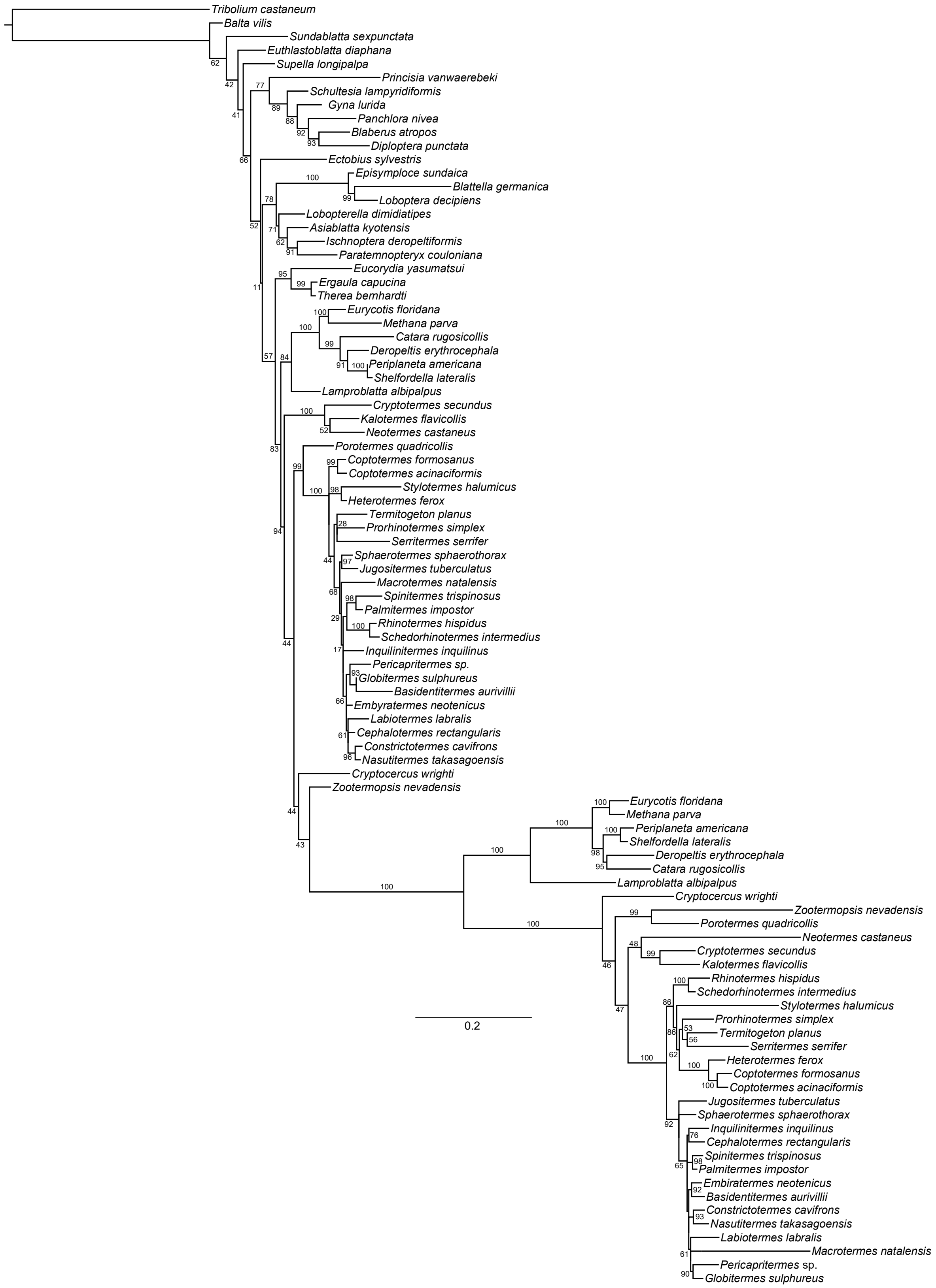

Supplementary Figure 8. Detailed phylogeny of FAD-A1 sequences identified in 57 members of Blattodea (28 termites and 29 cockroach species) and *T. castaneum* as an outgroup. The tree represents a full version of the simplified tree given in fig. 6 of the main text. The topology and branching supports were inferred using IQTREE maximum likelihood algorithm with LG+R7 model. The bootstrap values were calculated using ultrafast bootstrap approximation (UFBoot) from 1000 replicates. List of studied species and accession numbers of the analyzed FAD sequences are provided in supplementary table 4.

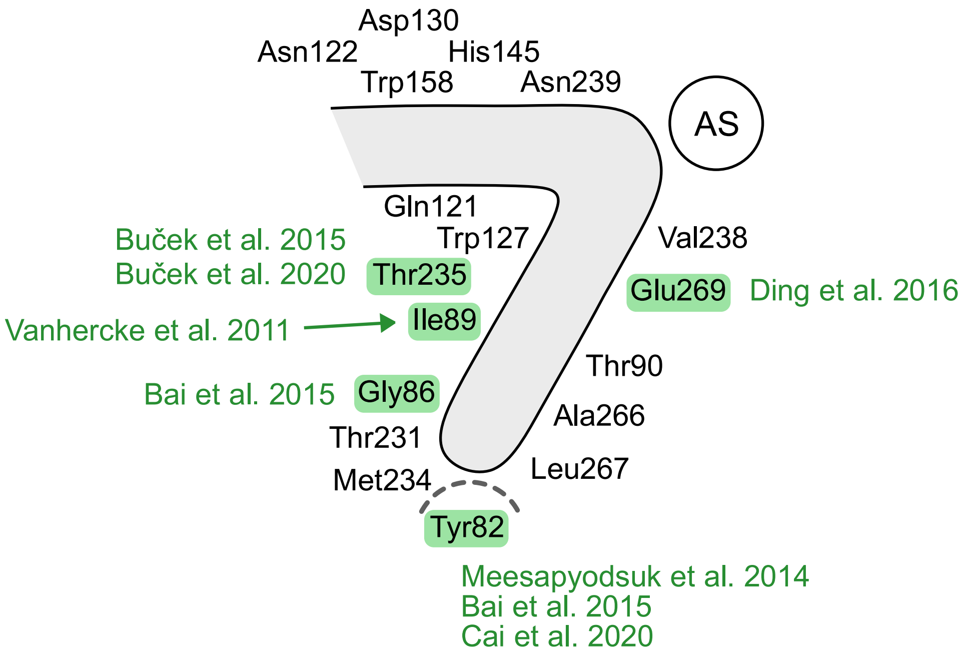

Supplementary Figure 9. Specificity determinants in the substrate tunnel. A diagram of *Psim*FAD-A1a substrate tunnel. The residues structurally homologous to those in *Psim*FAD-A1a which have been reported to influence FADs in regard to regiospecificity (Vanhercke et al. 2011; Buček et al. 2015; Buček et al. 2020), stereospecificity (Ding et al. 2016) and chain length preference (Vanhercke et al. 2011; Meesapyodsuk and Qiu 2014; Bai et al. 2015; Cai et al. 2020) are marked in green color.

Supplementary Table 1. List of 23 insect species used for the phylogenetic analysis of FAD sequences shown in fig. 1 of the main text and accession numbers of FAD sequences.

Abbreviation	Species	Order	Accession	Source	FAD family
**Edan**	*Ephemera danica*	Ephemeroptera	KAF4528972.1	GenBank	A1
			KAF4518902.1	GenBank	A2
			KAF4528974.1	GenBank	B
			KAF4526443.1	GenBank	C
			KAF4518903.1	GenBank	D
**Adom**	*Acheta domesticus*	Orthoptera	B7SB91	Uniprot	A1
			GDVN01018983.1	TSA	A1
			Q9BH41	Uniprot	A1
			GDVN01022114.1	TSA	B
			GDVN01049659.1	TSA	B
			GDVN01043610.1	TSA	C
			GDVN01021445.1	TSA	D
**Gbim**	*Gryllus bimaculatus*	Orthoptera	GFMG02075580.1	TSA	A1
			GFMG02139184.1	TSA	A1
			GFMG02141468.1	TSA	A1
			BOPP01000317.1	WGS	A2
			GFMG02050971.1	TSA	B
			GFMG02016204.1	TSA	B
			GFMG02063360.1	TSA	B
			BOPP01000093.1	WGS	C
			GFMG02047034.1	TSA	D
			BOPP01000015.1	WGS	E
**Same**	*Schistocerca americana*	Orthoptera	XP_046989329.1	GenBank	A1
			XP_046988300.1	GenBank	A2
			XP_046990268.1	GenBank	A2
			XP_046990306.1	GenBank	B
			XP_046998022.1	GenBank	C
			XP_046990422.1	GenBank	D
**Tshe**	*Timema shepardi*	Phasmatodea	GFPY01016024.1	TSA	A1
			GFPY01009484.1	TSA	A2
			GFPY01022877.1	TSA	B
			GFPY01020132.1	TSA	D
			GFPY01023923.1	TSA	E
**Bger**	*Blattella germanica*	Blattodea	PSN35395.1	GenBank	A1
			PSN54673.1	GenBank	A2
			PSN55622.1	GenBank	A2
			PSN37898.1	GenBank	A2
			PSN35396.1	GenBank	B
			PSN55191.1	GenBank	B
			PSN55195.1	GenBank	B
			PSN49612.1	GenBank	B
			PSN55142.1	GenBank	B
			PSN57577.1	GenBank	C
			PSN55599.1	GenBank	D
			PSN55637.1	GenBank	E
**Cwri**	*Cryptocercus wrighti*	Blattodea	GAZN02040058.1	TSA	A1
			GAZN02048323.1	TSA	A1
			GAZN02029622.1	TSA	A2
			GAZN02036641.1	TSA	A2
			GAZN02025943.1	TSA	B
			GAZN02048661.1	TSA	D
			GAZN02054823.1	TSA	D
			GAZN02036389.1	TSA	E
**Eneo**	*Embiratermes neotenicus*	Blattodea	OP575970	GenBank	A1
			OP575969	GenBank	A1
			OQ266415	GenBank	A2
			OQ266412	GenBank	B
			OQ266414	GenBank	C
			OQ266413	GenBank	D
			OQ266416	GenBank	E
**Iinq**	*Inquilinitermes inquilinus*	Blattodea	OQ266426	GenBank	A1
			OQ266425	GenBank	A1
			OQ266431	GenBank	A2
			OQ266427	GenBank	B
			OQ266429	GenBank	B
			OQ266430	GenBank	C
			OQ266428	GenBank	D
			OQ266432	GenBank	E

Supplementary Table 1 (continued). List of 23 insect species used for the phylogenetic analysis of FAD sequences shown in fig. 1 of the main text and accession numbers of FAD sequences.

Abbreviation	Species	Order	Accession	Source	FAD family
**Pame**	*Periplaneta americana*	Blattodea	OP575966	GenBank	A1
			OP575965	GenBank	A1
			GEIF01024354.1	TSA	A2
			PGRX01001866.1	WGS	A2
			PGRX01002240.1	WGS	B
			PGRX01009694.1	WGS	B
			GEIF01043641.1 + GEIF01030175.1	TSA	B
			GEIF01012415.1	TSA	D
			PGRX01001471.1	WGS	E
**Psim**	*Prorhinotermes simplex*	Blattodea	OP575964	GenBank	A1
			OP575963	GenBank	A1
			OQ266409	GenBank	A2
			OQ266410	GenBank	A2
			OQ266404	GenBank	B
			OQ266406	GenBank	B
			OQ266407	GenBank	B
			OQ266408	GenBank	C
			OQ266405	GenBank	D
			OQ266411	GenBank	E
**Rspe**	*Reticulitermes speratus*	Blattodea	RS006876	DOI: 10.6084/m9.figshare.c.5483235.v1	A1
			RS013686 + RS013687	DOI: 10.6084/m9.figshare.c.5483235.v1	A1
			RS010210	DOI: 10.6084/m9.figshare.c.5483235.v1	A2
			RS010208	DOI: 10.6084/m9.figshare.c.5483235.v1	A2
			RS006877	DOI: 10.6084/m9.figshare.c.5483235.v1	B
			RS006873	DOI: 10.6084/m9.figshare.c.5483235.v1	C
			RS006871	DOI: 10.6084/m9.figshare.c.5483235.v1	C
			RS010209	DOI: 10.6084/m9.figshare.c.5483235.v1	D
			RS001595	DOI: 10.6084/m9.figshare.c.5483235.v1	E
**Stri**	*Spinitermes trispinosus*	Blattodea	OQ266418	GenBank	A1
			OQ266417	GenBank	A1
			OQ266423	GenBank	A2
			OQ266419	GenBank	B
			OQ266421	GenBank	B
			OQ266422	GenBank	C
			OQ266420	GenBank	D
			OQ266424	GenBank	E
**Znev**	*Zootermopsis nevadensis*	Blattodea	OP575968	GenBank	A1
			OP575967	GenBank	A1
			XP_021921654.1	GenBank	A2
			XP_021941111.1	GenBank	A2
			XP_021939095.1	GenBank	B
			XP_021924158.1	GenBank	B
			XP_021924122.1	GenBank	B
			XP_021937379.1	GenBank	C
			XP_021937214.1	GenBank	D
			XP_021921664.1	GenBank	E
**Apis**	*Acyrthosiphon pisum*	Hemiptera	NP_001119674.1	GenBank	A1
			NP_001191886.1	GenBank	A1
			XP_001944248.1	GenBank	A1
			XP_001943726.1	GenBank	A2
			XP_008186444.1	GenBank	A2
			NP_001191869.1	GenBank	B
			XP_003247697.1	GenBank	B
			XP_029345352.1	GenBank	C
			XP_001943917.1	GenBank	X
**Mper**	*Myzus persicae*	Hemiptera	XP_022166482.1	GenBank	A1
			XP_022167133.1	GenBank	A1
			XP_022168655.1	GenBank	A2
			XP_022183415.1	GenBank	A2
			XP_022168642.1	GenBank	B
			XP_022168650.1	GenBank	B
			XP_022172376.1	GenBank	C
			XP_022166479.1	GenBank	X
**Amel**	*Apis mellifera*	Hymenoptera	XP_026295893.1	GenBank	A1
			XP_026296155.1	GenBank	A2
			XP_026296165.1	GenBank	A2
			XP_006562277.2	GenBank	B
			XP_026296160.1	GenBank	D
			XP_026295891.1	GenBank	E

Supplementary Table 1 (continued). List of 23 insect species used for the phylogenetic analysis of FAD sequences shown in Fig. 1 of the main text and accession numbers of FAD sequences.

Abbreviation	Species	Order	Accession	Source	FAD family
**Nvit**	*Nasonia vitripennis*	Hymenoptera	XP_003425691.1	GenBank	A1
			XP_031783782.1	GenBank	A1
			XP_008205135.1	GenBank	A2
			XP_001599873.1	GenBank	B
			XP_001599877.2	GenBank	B
			XP_001602540.1	GenBank	B
			XP_001607533.2	GenBank	B
			XP_001599579.1	GenBank	E
**Clug**	*Chauliognathus lugubris*	Coleoptera	AFJ66830.1	GenBank	A1
			AFJ66831.1	GenBank	A1
			AFJ66833.1	GenBank	A1
			AFJ66829.1	GenBank	A1
			AFJ66832.1	GenBank	A1
			AFJ66827.1	GenBank	A1
			AFJ66828.1	GenBank	A1
**Cmac**	*Callosobruchus maculatus*	Coleoptera	VEN53100.1	GenBank	A1
			VEN36853.1	GenBank	A2
			VEN33644.1	GenBank	B
			VEN33646.1	GenBank	B
			VEN64464.1	GenBank	C
			VEN50495.1	GenBank	D
**Tcas**	*Tribolium castaneum*	Coleoptera	ADK13054.1	GenBank	A1
			ADK13055.1	GenBank	A1
			AHH30808.1	GenBank	A2
			AHH30809.1	GenBank	A2
			AHH30810.1	GenBank	A2
			AHH30811.1	GenBank	A2
			ABY26958.1	GenBank	A2
			AHH30813.1	GenBank	A2
			AHH30815.1	GenBank	A2
			AHH30812.1	GenBank	C
			AHH30814.1	GenBank	D
**Bmor**	*Bombyx mori*	Lepidoptera	NP_001036914.1	GenBank	A1
			NP_001036971.1	GenBank	A1
			NP_001037017.2	GenBank	A1
			NP_001037018.1	GenBank	A1
			NP_001040141.1	GenBank	A1
			NP_001274330.1	GenBank	A1
			NP_001296477.1	GenBank	A1
			NP_001296508.1	GenBank	A1
			XP_004925565.1	GenBank	A2
			NP_001296494.1	GenBank	B
			XP_021203267.2	GenBank	B
			XP_037867672.1	GenBank	B
			NP_001296530.1	GenBank	C
			NP_001296505.1	GenBank	D
**Msex**	*Manduca sexta*	Lepidoptera	XP_030022752.1	GenBank	A1
			XP_030022753.1	GenBank	A1
			XP_030028979.2	GenBank	A1
			XP_030030645.1	GenBank	A1
			XP_030032153.1	GenBank	A1
			XP_030033583.2	GenBank	A1
			XP_030034931.2	GenBank	A1
			XP_037298899.1	GenBank	A1
			XP_037299299.1	GenBank	A1
			XP_037298044.1	GenBank	A2
			XP_030020161.1	GenBank	B
			XP_030033763.1	GenBank	B
			XP_030033888.1	GenBank	B
			XP_030036120.1	GenBank	C
			XP_037298041.1	GenBank	D

Supplementary Table 2. Peak areas of FAMEs from functional assays with *Psim*FAD-A1a and its mutants, used to calculate the conversion rates of saturated FAs (stearic acid, palmitic acid, myristic acid) into *Z*9-monounsaturated homologs. The table is related to fig. 4 of the main text.

Compound	Peak area						
	control	wt1	wt2	Gly86Ile1	Gly86Ile2	Trp236Leu1	Trp236Leu2
9Z-14:1	0	2037120	3438499	9427996	9368875	0	0
14:0	48255409	37982275	63901761	46633927	52355298	44062867	1597729
9Z-16:1	0	213028104	163178983	3161490	3271121	5060411	7012041
16:0	686498408	498566633	423319425	386420306	362786828	407149700	364991429
9Z-18:1	0	757692373	976409466	2373891	1313235	21331989	971555
18:0	89221829	38000774	54915192	109099289	134603844	93016651	4767062

Supplementary Table 3. Expression of FAD-A1a and A1b in four castes and two tissues of the termite *P. simplex*. Data represent FPKM (fragments per kilobase million) estimated from RNA Seq analysis. The table is related to fig. 5 of the main text.

	FAD-A1a (FPKM)	
caste	head	abdomen
worker	142.8	135.99
	62.58	574.13
	110.29	311.81
soldier	79.06	106.89
	79.56	434.68
	101.75	117.7
primary king	159.93	350.76
	203.39	269.33
primary queen	131.27	124.52
	415.13	273.29
	269.82	228.04

	FAD-A1b (FPKM)	
caste	head	abdomen
worker	11.01	7.16
	12.01	39.33
	8.02	10.9
soldier	3.11	9.04
	3.82	20.52
	1.1	10.66
primary king	4.73	8.88
	6.18	13.78
primary queen	11.27	20.71
	28.53	21.32
	9.56	11.7

Supplementary Table 4. List of cockroach and termite species used for the detailed phylogenetic analysis of FAD-A1 sequences identified in 57 Blattodea (28 termites and 29 cockroach species) and their accession numbers. Taxonomy is used according to Evangelista et al. (2019) for cockroaches and Buček et al. (2019) for termites. The table is related to fig. 6 of the main text.

Abbreviation	Species	Order	Clade	Subclade	Family	Subfamily	Accession number	
							FAD-A1a	FAD-A1b
**Tcas**	*Tribolium castaneum*	Coleoptera	Cucujiformia	Tenebrionoidea	Tenebrionidae		ADK13054.1	–
**Akyo**	*Asiablatta kyotensis*	Blattodea	Blaberoidea		Ectobiidae	Blattellinae	GDWW01040879.1	–
**Bger**	*Blattella germanica*	Blattodea	Blaberoidea		Ectobiidae	Blattellinae	PSN35395.1	–
**Bvil**	*Balta vilis*	Blattodea	Blaberoidea		Ectobiidae	Pseudophyllodromiinae	GDYJ01096141.1	–
**Edia**	*Euthlastoblatta diaphana*	Blattodea	Blaberoidea		Ectobiidae	Pseudophyllodromiinae	GDZY01059099.1	–
**Esun**	*Episymploce sundaica*	Blattodea	Blaberoidea		Ectobiidae	Blattellinae	GDYC01052721.1	–
**Esyl**	*Ectobius sylvestris*	Blattodea	Blaberoidea		Ectobiidae	Ectobiinae	GDYP01021383.1	–
**Ider**	*Ischnoptera deropeltiformis*	Blattodea	Blaberoidea		Ectobiidae	Blattellinae	GDEC01032119.1	–
**Ldec**	*Loboptera decipiens*	Blattodea	Blaberoidea		Ectobiidae	Blattellinae	GDYK01062559.1	–
**Ldim**	*Lobopterella dimidiatipes*	Blattodea	Blaberoidea		Ectobiidae	Blattellinae	GDZZ01023908.1	–
**Pcou**	*Paratemnopteryx couloniana*	Blattodea	Blaberoidea		Ectobiidae	Blattellinae	GDZI01050018.1	–
**Slon**	*Supella longipalpa*	Blattodea	Blaberoidea		Ectobiidae	Pseudophyllodromiinae	GDWU01053106.1	–
**Ssex**	*Sundablatta sexpunctata*	Blattodea	Blaberoidea		Ectobiidae	Pseudophyllodromiinae	GDCJ01058588.1	–
**Batr**	*Blaberus atropos*	Blattodea	Blaberoidea		Blaberidae		GAYD02052627.1	–
**Dpun**	*Diploptera punctata*	Blattodea	Blaberoidea		Blaberidae		GDYG01011972.1	–
**Glur**	*Gyna lurida*	Blattodea	Blaberoidea		Blaberidae		GDZJ01015375.1	–
**Pniv**	*Panchlora nivea*	Blattodea	Blaberoidea		Blaberidae		GDWQ01049945.1	–
**Pvan**	*Princisia vanwaerebeki*	Blattodea	Blaberoidea		Blaberidae		GDXG01037189.1	–
**Slam**	*Schultesia lampyridiformis*	Blattodea	Blaberoidea		Blaberidae		GCPR01062913.1	–
**Ecap**	*Ergaula capucina*	Blattodea	Solumblattodea	Corydioidea	Corydiidae		GDBS01086068.1	–
**Eyas**	*Eucorydia yasumatsui*	Blattodea	Solumblattodea	Corydioidea	Corydiidae		GDZF01072797.1	–
**Tber**	*Therea bernhardti*	Blattodea	Solumblattodea	Corydioidea	Corydiidae		GDVQ01048815.1	–
**Crug**	*Catara rugosicollis*	Blattodea	Solumblattodea	Blattoidea	Blattidae		GDCA01034883.1	GDCA01017170.1
**Dery**	*Deropeltis erythrocephala*	Blattodea	Solumblattodea	Blattoidea	Blattidae		GDZH01056164.1	GDZH01051688.1
**Eflo**	*Eurycotis floridana*	Blattodea	Solumblattodea	Blattoidea	Blattidae		GDYS01051098.1	GDYS01034590.1
**Mpar**	*Methana parva*	Blattodea	Solumblattodea	Blattoidea	Blattidae		GDWT01039622.1	GDWT01027380.1
**Pame**	*Periplaneta americana*	Blattodea	Solumblattodea	Blattoidea	Blattidae		GAWS02041257.1	GAWS02044430.1
**Slat**	*Shelfordella lateralis*	Blattodea	Solumblattodea	Blattoidea	Blattidae		GDXP01061090.1	GDXP01059289.1
**Lalb**	*Lamproblatta albipalpus*	Blattodea	Solumblattodea	Blattoidea	Lamproblattidae		GCPS01047675.1	GCPS01045818.1
**Cwri**	*Cryptocercus wrighti*	Blattodea	Solumblattodea	Blattoidea	Cryptocercidae		GAZN02040058.1	GAZN02048323.1
**Znev**	*Zootermopsis nevadensis*	Blattodea	Solumblattodea	Blattoidea: Isoptera	Archotermopsidae		XP_021939090.1	XP_021926395.1
**Pqua**	*Porotermes quadricollis*	Blattodea	Solumblattodea	Blattoidea: Isoptera	Stolotermitidae		GIAG01025063.1	GIAG01112714.1
**Csec**	*Cryptotermes secundus*	Blattodea	Solumblattodea	Blattoidea: Isoptera	Kalotermitidae		XP_023717587.1	XP_023727613.1
**Kfla**	*Kalotermes flavicollis*	Blattodea	Solumblattodea	Blattoidea: Isoptera	Kalotermitidae		GHWY01057140.1	GHWY01164215.1
**Ncas**	*Neotermes castaneus*	Blattodea	Solumblattodea	Blattoidea: Isoptera	Kalotermitidae		GIAC01022370.1	GIAC01022369.1
**Shal**	*Stylotermes halumicus*	Blattodea	Solumblattodea	Blattoidea: Isoptera	Stylotermitidae		GIAK01072481.1	GIAK01038699.1
**Rhis**	*Rhinotermes hispidus*	Blattodea	Solumblattodea	Blattoidea: Isoptera	Rhinotermitidae	Rhinotermitinae	GIAL01156603.1	GIAL01156598.1
**Sint**	*Schedorhinotermes intermedius*	Blattodea	Solumblattodea	Blattoidea: Isoptera	Rhinotermitidae	Rhinotermitinae	GIAM01215096.1	GIAM01139546.1
**Psim**	*Prorhinotermes simplex*	Blattodea	Solumblattodea	Blattoidea: Isoptera	Rhinotermitidae	Prorhinotermitinae	OP575964	OP575963
**Tpla**	*Termitogeton planus*	Blattodea	Solumblattodea	Blattoidea: Isoptera	Rhinotermitidae	Termitogetoninae	GHXB01034336.1	GHXB01037344.1
**Sser**	*Serritermes serrifer*	Blattodea	Solumblattodea	Blattoidea: Isoptera	Serritermitidae		GIAH01057750.1	GIAH01074852.1
**Caci**	*Coptotermes acinaciformis*	Blattodea	Solumblattodea	Blattoidea: Isoptera	Rhinotermitidae	Coptotermitinae	GHZJ01191630.1	GHZJ01114992.1
**Cfor**	*Coptotermes formosanus*	Blattodea	Solumblattodea	Blattoidea: Isoptera	Rhinotermitidae	Coptotermitinae	GFG32026.1	GFG40379.1
**Hfer**	*Heterotermes ferox*	Blattodea	Solumblattodea	Blattoidea: Isoptera	Rhinotermitidae	Heterotermitinae	GHZZ01430969.1	GHZZ01290703.1
**Mnat**	*Macrotermes natalensis*	Blattodea	Solumblattodea	Blattoidea: Isoptera	Termitidae	Macrotermitinae	Mnat-01743*	Mnat-08601*
**Ssph**	*Sphaerotermes sphaerothorax*	Blattodea	Solumblattodea	Blattoidea: Isoptera	Termitidae	Sphaerotermitinae	GIAI01021156.1	GIAI01050698.1
**Jtub**	*Jugositermes tuberculatus*	Blattodea	Solumblattodea	Blattoidea: Isoptera	Termitidae	Apicotermitinae	GHZX01030840.1	GHZX01069879.1
**Eneo**	*Embiratermes neotenicus*	Blattodea	Solumblattodea	Blattoidea: Isoptera	Termitidae	Syntermitinae	OP575970	OP575969
**Llab**	*Labiotermes labralis*	Blattodea	Solumblattodea	Blattoidea: Isoptera	Termitidae	Syntermitinae	GIAA01080485.1	GIAA01174380.1
**Ccav**	*Constrictotermes cavifrons*	Blattodea	Solumblattodea	Blattoidea: Isoptera	Termitidae	Nasutitermitinae	GHZF01205521.1	GHZF01185299.1
**Ntak**	*Nasutitermes takasagoensis*	Blattodea	Solumblattodea	Blattoidea: Isoptera	Termitidae	Nasutitermitinae	IAEB01028719.1	IAEB01001592.1
**Baur**	*Basidentitermes aurivillii*	Blattodea	Solumblattodea	Blattoidea: Isoptera	Termitidae	Cubitermitinae	GHZB01207929.1	GHZB01283038.1
**Crec**	*Cephalotermes rectangularis*	Blattodea	Solumblattodea	Blattoidea: Isoptera	Termitidae	Termitinae	GHZD01116669.1	GHZD01046330.1
**Gsul**	*Globitermes sulphureus*	Blattodea	Solumblattodea	Blattoidea: Isoptera	Termitidae	Termitinae	GHZP01061940.1	GHZP01023886.1
**Iinq**	*Inquilinitermes inquilinus*	Blattodea	Solumblattodea	Blattoidea: Isoptera	Termitidae	Termitinae	OQ266426	OQ266425
**Pimp**	*Palmitermes impostor*	Blattodea	Solumblattodea	Blattoidea: Isoptera	Termitidae	Termitinae	GIAD01119611.1	GIAD01140999.1
**Pspe**	*Pericapritermes sp.*	Blattodea	Solumblattodea	Blattoidea: Isoptera	Termitidae	Termitinae	GIAE01023751.1	GIAE01143473.1
**Stri**	*Spinitermes trispinosus*	Blattodea	Solumblattodea	Blattoidea: Isoptera	Termitidae	Termitinae	OQ266418	OQ266417

Supplementary Table 5. Summary of substitutions accumulated during the evolution of FAD-A1b. In red bold font residues discussed in the main text, those with expected functional significance marked with exclamation mark. Substitutions distinguishing FAD-A1b of Blattidae + Lamproblattidae from termites + *Cryptocercus* are marked with orange shading.

Substrate tunnel interior

expected functional importance	position (relative to PsimFAD-A1a)	FAD	dominant residue (incidence)	alternative residues
	82	A1a	Tyr (57/57)	—
		A1b	Tyr (32/33)	1 Phe
**!**	**86**	**A1a**	**Gly (56/57)**	**1 Met**
		**A1b**	**Ile (31/32)**	**1 Val**
	89	A1a	Ile (53/57)	1 Val
		A1b	Ile (31/34)	1 Val, 1 Ala, 1 Asn
	90	A1a+b	Thr (91/91)	—
	121	A1a+b	Gln (92/92)	—
	**122**	**A1a**	**Asn (57/57)**	**—**
		**A1b**	**Thr (30/35)**	**3 Met, 2 Asp**
	127	A1a+b	Trp (93/93)	—
	**130**	**A1a**	**Asp (57/57)**	**—**
		**A1b**	**Asn (36/36)**	**—**
	145	A1a+b	His (93/93)	—
	158	A1a+b	Trp (93/93)	—
	235	A1a	Thr (54/57)	3 Ser
		A1b	Thr (35/36)	1 Ala
**!**	**236**	**A1a**	**Trp (57/57)**	**—**
		**A1b**	**Leu (36/36)**	**—**
	**238**	**A1a**	**Val (56/56)**	**—**
		**A1b**	**Val (7/7 in Blattodea + Lamproblattidae)**	**—**
			**Thr (27/29 in Isoptera + *Cryptocercus*)**	**1 Ser, 1 Phe**
	231	A1a	Thr (57/57)	—
		A1b	Thr (35/36)	1 Ala
	234	A1a	Met (45/57)	4 Thr, 3 Ala, 3 Ile, 2 Val
		A1b	Met (33/36)	2 Ile, 1 Ala
	239	A1a+b	Asn (93/93)	—
	**266**	**A1a**	**Ala (57/57)**	**—**
		**A1b**	**Thr (35/36)**	**1 Ser**
	267	A1a	Leu (56/57)	1 Met
		A1b	Leu (36/36)	—
	269	A1a+b	Glu (93/93)	—

Substrate tunnel exterior

expected functional importance	position (relative to PsimFAD-A1a)	FAD	dominant residue (incidence)	alternative residues
	**47**	**A1a**	**Arg (56/57)**	**1 Ile**
		**A1b**	**Pro (29/33)**	**2 Leu, Arg, Ser**
	48	A1a	Asn (56/57)	1 Val
		A1b	Asn (32/33)	1 Tyr
	**51**	**A1a**	**Leu (56/56)**	**—**
		**A1b**	**Met (24/33)**	**5 Leu, 3 Val, Gly**
	**52**	**A1a**	**Phe (56/56)**	**—**
		**A1b**	**His (33/33)**	**—**
	55	A1a	Leu (56/56)	—
		A1b	Leu (28/33)	4 Met, 1 Phe
	120	A1a+b	Phe (92/92)	—
	**159**	**A1a**	**Leu (57/57)**	**—**
		**A1b**	**Met (34/36)**	**1 Ile, 1 Val**
	**228**	**A1a**	**Tyr (57/57)**	**—**
		**A1b**	**His (7/7 in Blattodea + Lamproblattidae)**	**—**
			**Gln (28/29 in Isoptera + *Cryptocercus*)**	**1 His**
	232	A1a+b	93/93 Leu	—

Supplementary Table 6. List of FAD gene constructs used for heterologous expression and functional characterization assays. Sequences originating from pYEXTHS-BN vector marked in red.

Gene	Sequence
**PsimFAD-A1a**	ATGTCTCATCACCATCACCATCACGGATCCATGGCTCCAAATATTACTTCTTCACCAACAGGTGTTTTGTTCGAAGATGATATTGTTGAAACTGTTGCAGTTTCTTCAGTTGAACCTAAAA<br>ATCATGATTCTAAGCCACCACAAAAGTACTCAAGACAAATCGTTTGGAGAAACGTTATCTTGTTCGTTTATTTGCATTTGGCTGCAGTTTATGGTGCTTACTTAATGTTGACTTCTGCAAA<br>GATCATCACTACAGTTTTCGCTGTTTTGTTATACCAAGCAGGTGGTTTAGGTATTACAGCTGGTGCACATAGATTGTGGGCTCATAGATCATACAAGGCAAAGTGGCCATTGAGATTGTTA<br>TTGGTTATTTTTAATACTTTGGCTTTTCAAAATGATGTTTGTGAATGGGCAAGAGATCATAGAGTTCATCATAAATTTTCTGAAACTGATGCTGATCCACATAATGCAACTAGAGGTTTCT<br>TTTTCTCTCATGTTGGTTGGTTATTGGTTAGAAAGCATCCAGATGTTAAGGAAAAGGGTAAAACAATCGATATGTCTGATTTGGAAGCTGATCCATTTTTGAGATTCCAAAAGAAATACTA<br>CTTATTGGTTATGCCATTGTTGTGTTTCGTTTTGCCAACTTTGGTTCCAGTTTACTTATGGGGTGAAACATGGTCTAATGCTTGGTTTGTTGTTGCAATGTTCAGATACACTTTTACATTG<br>AACATGACTTGGTTGGTTAATTCAGCTGCACATTTTTGGGGTAACAGACCATACGATAAGTACATCAACCCAGCTGAAAATTTGGGTGTTGCTGTTTTTGCATTGGGTGAAGGTTGGCATA<br>ACTACCATCATGTTTTCCCTTGGGATTACAAGACTGCAGAATTAGGCAACTACAGAACAAATTTGACTACAGCTTTTATTGATTTCTTTTCAAGAATTGGTTGGGCTTATGATTTGAAGAC<br>TGTTCCATTGTCAATGGTTAAAAGAAGAGTTGAAAGAACAGGTGACGGTTCTCATGAAGTTTGGGGTTGGGGTGACAAAGATATGTCACAAGAAGATATGGATGAAGCTCAAGTTATTAAT<br>AAGAAAGTTACATAA
**PsimFAD-A1b**	ATGTCTCATCACCATCACCATCACGGATCCATGGCTCCAAATGTAACAAGTTCTCCCACTGAAGTTTTGTGCACCCCAGAAACCACACTACATTCGTCAAATGAAATAAAGGATGGAATCG<br>AAAAACCAGCCCTGAAGTACAGAAGAGAAATTGTGTGGCCAAATGTTGTCATGCATATTTATTTGAATTTGGCGGCGCTTTATGGAGCCTACATGATGTTGACATCTGCTAAACTACTGAC<br>GGGATTATGGGCCTTTATTCTGTATGAAGGAGGAATACTGGGGATCACAGCTGGTGCACACAGACTGTGGTCGCACCGTGCTTACAGGGCCACTTGGCAGTTGCGCCTGATTCTTATGCTT<br>CTGCAGACCCTGGCTTTCCAGATGTCTGTGCACGAGTGGGTTCGTAACCACAGGGTGCACCACAAAAACAGCGATACGGATGCTGACCCGCATAACGTGCGCCGTGGTTTCTTCTTCACTC<br>ATTACGGCTGGATGTTAGTACGAAAACATCCGGCAGTGAAGGAAAAAGGGAAGAAAATCGACTGGTCAGATCTGGATGCGGACGCGTTGCTGGTGTTCCAAAAGAGGTACTATATCATTTT<br>AATGCCAGTTTTGTGCTTCCTGATACCAAGTGCAGTCCCTGTGTATCTGTGGGGTGAGACGTGGACGAATGCGTATCACATCGCTGCTGTTTTGCGTCAAGTAGTCACACTTCACATGACA<br>CTTATCACTAACAGTATTACACATTGGCCAGACACGTGGAGAAAACGACCCTATGACAAGAATATTTTTCCATCAGAGAACTTCATGGTCTCATTGTTAACTCTTGGAGAAGGTTGGCATA<br>ATTTTCACCATGTATTTCCGTGGGATTATAAAACTGCTGAAATCGGAGGTAACCGCATTAACCCCACTACGCGCTTTATTGACTTCTGTGCTCGCATCGGATGGGCGTACGACCTGAAGAC<br>AGTTCCCATGTCCGTGGTGAAGAGGCGAATAGAACGCACTGGGGACGGGACTCATGAAATCTGGGGCTGGGGTGACAAAGACATGAGCACAAGAGACAAGAGTCTGGTAGAAGTCGTCAAT<br>CAGGAAGCGAAGTGA
**PameFAD-A1a**	ATGTCTCATCACCATCACCATCACGGATCCATGGCTCCAAATATTACTTCTTCACCAACAGGTGTTTTGTTCGAAGATGATACTATCGAAACTGTTACATTGCCAACAATCGAAACTAAGA<br>CAGATGATTCTAAGCCAGCAGAAAAGTACAAGAGAAAGATCGTTTGGAGAAACGTTATCTTGTTTGTTTATTTGCACATGGCTGCATTATATGGTGCTTACTTGATGTTGACTTCTTGTAA<br>ATTGATCACAGCTATTTGGGCAATCTTGTTGTACCAAGCAGGTGGTTTAGGTATTACTGCTGGTGCACATAGATTGTGGTCACATAGAGCTTACAAAGCAAAATGGCCATTGAGATTGATT<br>TTGGTTATTTTTAATACTTTAGCTTTTCAAAATCATGTTTATGAATGGGCAAGAGATCATAGAGTTCATCATAAATTTTCTGAAACAGATGCTGATCCACATAATGCAAAAAGAGGTTTCT<br>TTTTCTCTCATGTTGGTTGGTTGTTAGTTAGAAAGCATCCAGATGTTAAGGTTAAGGGTAAAGGTATCGATATGTCTGATTTGGATGCTGATCCATTGATCGCATTCCAAAAGAAACATTA<br>CTTAATTTTGATGCCAATTATTTGTTTCATCTTGCCAACTATCATTCCAGTTTATTTTTGGGGTGAAACATGGTCTAATGCTTGGTTCGTTGTTGCAATGTTCAGATACACTTTTACATTG<br>AACGCTTCTTGGTTGGTTAATTCAGCTGCACACATGTGGGGTTCAAGACCATACGATAAGTACATCAACCCATCTGAAAATTTGGGTGTTTCAATGTTAGCTTTGGGTGAAGGTTGGCATA<br>ACTACCATCATGTTTTCCCTTGGGATTACAAGACTGCAGAATTAGGCAACTACTCTACAAATTTGACTACAGCTTTTATTGATTTCTTTTCAAGAATTGGTTGGGCTTATGATTTGAAGAC<br>TGTTCCAATGTCAATGGTTAAACAAAGAGTTCAAAGAACAGGTGACGGTTCTCATGATGTTTGGGGTTGGGGTGACAAAGATATGTCACAAGAAGATATGGATGAAGCTCAAGTTATTAAT<br>AAGAAATTGAAATAA
**PameFAD-A1b**	ATGTCTCATCACCATCACCATCACGGATCCATGGCACCAAATATTACTTCTACACCAACTGGTGTTTTGTATGAAGAAGATTTTGCTGCAGCTGAAAAAGCTACATCAACTGAAACAAAGG<br>AAGGTATTAAACCAAAGAGAGAATACAAGAAACAAATCGTTTGGCCAAACGTTATCATGCATTTCTTGTTGCATGTTGGTGCAGTTTATGGTGCTTACTTGATGTTGACTTCTGCAAAAAT<br>TTTGACAGGTATTTGGGCTTTCTTTTTGTACGAAGTTGGTATTTTAGGTATCACTGCAGGTGCTCATAGATTATGGTCTCATAGATCATACAAAGCAACATGGCAAATGAGATTGATCTTG<br>ATGATCTGTCAAACTGTTTCTTTTCAAACATCAGTTCATGAATGGGCTAGAAACCATAGAGTTCATCATAAGCATTCTGATACTGATGGTGACCCACATAACGTTAACAGAGGTTTGTTTT<br>TCTCTCATGCAGGTTGGATGATGTGTAGAAAGCATCCAGAAGTTAAGGAAAAGGGTAAACAAATTGATTTGTCTGATTTGGATGCTGATCCAATCTTGATGTTCCAAAAGAAATATTACTT<br>GATTTTAATGCCTTTTATGTGTTTCTTTTTGCCAACTTGGATTCCAGTTTATTTTTGGGGTGAAACATGGCATAACGCTTACTTCGTTGCAGCTATTTTTAGACATGTTTTTACTTTGAAC<br>ATGACATTAATGGTTAATTCTATCACTCATAACACATGGGGTAACAGACCATACGATAAGAACATCAACCCAGCAGAAAACGCTATCGTTTCATTGATGACTTTAGGTGAAGGTTGGCATA<br>ACTACCATCATGTTTTCCCTTGGGATTACAAAACAGCAGAATTGGGTGTTTTGAGAATTAATATGACTACATTGTTTATTGATTTGTGTGCAAAGATTGGTTGGGCTTACGATTTGAAAAC<br>TGTTCCAATGGATATGGTTAAAAGAAGAGTTGAAAGAACTGGTGACGGTACACATGAAATTTGGGGTTGGGGTGACAAGGATATGACAGAAAAGGAAAGAGAAATCGCTCAAATTATTAAT<br>AAGAAAGATTAA
**ZnevFAD-A1a**	ATGTCTCATCACCATCACCATCACGGATCCATGGCACCAAATATTACTTCTTCACCAACAGGTGTTTTGTTCGAAGATGATATTGTTGAAACTGTTGCAGTTCCATCTATCGAATCTAAAA<br>ATGATGCTAAACCACCAGAAAAGTACAGAAGACAAATCGTTTGGAGAAACGTTATCTTGTTCGCTTATTTGCATTTGGCTGCAGTTTATGGTGCATACTTGATGTTGACTTCTGCTAAGAT<br>CATCACATCAATTTGGGCAATCTTGTTATACCAAGCTGGTGGTTTGGGTATTACAGCTGGTGCACATAGATTATGGGCTCATAGAGCATACAAGGCTAAGTGGCCATTGAGATTGATCTTG<br>ATGTTGTTTAATACTTTGGCATTTCAAAATCATGTTCATGAATGGGCTAGAGATCATAGAGTTCATCATAAATTTTCTGAAACAGATGCAGATCCACATGATGCTAGAAGAGGTTTCTTTT<br>TCTCTCATGTTGGTTGGTTGTTAGTTAGAAAGCATCCAGATGTTAAGGAAAAGGGTAAAGGTATCGATATGTCTGATTTGGATACTGATCCATTGTTGATGTTCCAAAAGAAATACTACTT<br>AATCATGATGCCAATTTTGTGTTTCTTGATTCCAACTATGGTTCCAGTTTACTTGTGGGGTGAATCATGGACAAATGCATGGTTTGTTGTTGCTATGTTCAGATACACTTTTACATTGAAC<br>ATGACTTGGTTGGTTAATTCTGCTGCACACATGTGGGGTTCACAACCATACGATAAGTACATCAACCCAGCTGAAAATTTGTCTGTTGCAGTTTTGGCTTTAGGTGAAGGTTGGCATAACT<br>ACCATCATGTTTTCCCTTGGGATTACAAGACAGCAGAATTGGGTAACTACAGAGCAAATTTGACTACAGCTTTTATTGATTTCTTTTCAAGAATTGGTTGGGCTTATGATTTGAAGACTGT<br>TCCAATGTCAATGGTTAAAAGAAGAGTTGAAAGAACAGGTGACGGTTCTCATGAAGTTTGGGGTTGGGGTGACAAAGATATGTCACAAGATGATATGGATCAAGCTCAAGTTATTAATAAT<br>AAGTCTAAATAA
**ZnevFAD-A1b**	ATGTCTCATCACCATCACCATCACGGATCCATGGCTCCAAATTCTTCATCTGTTACTGAAGATAGAGGTGCAGGTGACACAGCTCATGCACCACAAAATGAAAGAAGAGGTGCTGGTGCAG<br>AACCATATCCAAATTACAGAACTGAAGTTGTTTGGCCAAACGTTGTTGTTCATGCTTATTTGAATTTGGCTGCATTGTATGGTGCTTACTTAATGTTTACTTCAGCAAGATTGCCAACAGC<br>TATTTGGGCTTTTATTTTGTACGAAGCTGGTATTTTAGGTATTACTGCTGGTGCACATAGATTGTGGTCTCATAGAGCTTACAAAGCAACATGGCCATTGAGAGTTTTCTTGGTTTTGTTG<br>CAAACTTTAGCTTTCCAAACATCAGCACATGAATGGGTTAGAAACCATAGAGTTCATCATAAATTTTCTGATACTGATGCTGATCCACATAACGTTAACAGAGGTTTCTTTTTCACTCATT<br>ACGGTTGGATGTTGACTAAGAAACATCCAGCTGTTAAGGAAAACGGTAAAAAGGTTGATTCTTCTGATTTGGATGCAGATCCATTGTTGATCTTCCAAAAGAGATACTACATGATTATTAT<br>GCCAATCTTGTGTTTCGTTATCCCAACTGCTGTTCCAGTTTTTGTTTGGAACGAAACATGGTCAAACGCATACCATATCGCTGCAGTTATGAGACAAGTTATTACATTGCACATGACTTTG<br>ATCACAAATTCTGTTACTCATTGGCCAGATACATGGAGAAAGAGACCATACGATAAGAGAATTTGTCCATCAGAAAATTTCTTTGTTTCTTTGTTGACTTTGGGTGAAGGTTGGCATAACT<br>TCCATCATGTTTTCCCTTGGGATTACAAGACATCAGAAATCGGTTACAACAGAATTAATCCAACTACACATTTCATCGATTTCTGTGCTAGAATTGGTTTGGCTTATGATTTGAAGACTGT<br>TCCAACATCAATGGTTAAAAGAAGAGTTCAAAGAACTGGTGACGGTACACATGAATTATGGGGTTGGGGTGACGCTGATATGTCTGAAGCAGAAAAGAAAATGGTTCAAGTTGTTAACGAA<br>AAGGCTACATAA
**EneoFAD-A1a**	ATGTCTCATCACCATCACCATCACGGATCCATGGCTCCAAATATTACTGGTTCTCCAACAGGTGTTTTGTTCGAAGATGATACTGTTGAAACTGTTACAGTTCCATCTGTTGAACCTAAAA<br>ATCATGATTCAAAGCCACCAGAAAACTACAGAAGACAAATCGTTTGGAGAAACGTTATCTTGTTCGTTTATTTGCATGTTGCTGCAGTTTATGGTGCTTACTTGATGTTGACTTCAGCAAA<br>GATCATCACTACAGTTTGGGCTATCTTGTTATACCAAGCAGGTGGTTTAGGTATTACAGCTGGTGCACATAGATTGTGGGCTCATAGAGCATACAAGGCTAAGTGGCCATTGAGATTGATT<br>TTGGTTATTTTTAATACATTGGCTTTTCAAAATGATGTTTGTGAATGGGCAAGAGATCATAGAGTTCATCATAAATTTTCTGAAACTGATGCAGATCCACATAATGCTACTAGAGGTTTCT<br>TTTTCTCTCATGTTGGTTGGTTGTTAGTTAGAAAGCATCCAGATGTTAAGGAAAAGGGTAAAGCTATCGATATGTCTGATTTGGAAGCAGATCCATTTTTGAGATTCCAAAAGAAATACTA<br>CTTAATCGTTATGCCAATTTTATGTTTCTTGGTTCCAACTTGTGTTCCAGTTTACTTGTGGGGTGAAACATGGTCTAATGCATGGTTCGTTGTTGCTATGTTCAGATACGCTTTTACTTTG<br>AACATGACATGGTTGGTTAATTCAGCTGCACATATTTGGGGTACTAGACCATACGATAAGTACATCAACCCAGCTGAAAATTTGGGTGTTGCAGTTTTTGCTTTGGGTGAAGGTTGGCATA<br>ACTACCATCATGTTTTCCCTTGGGATTACAAGACAGCTGAATTAGGCAACTACAGAGCTAATTTGACTACAGCTTTTATTGATTTCTTTTCAAGAATTGGTTGGGCTTATGATTTGAAGAC<br>TGTTCCATTGTCAATGGTTAAAAGAAGAGTTGAAAGAACAGGTGACGGTTCTCATGAAGTTTGGGGTTGGGGTGACAAAGATATGTCACAAGAAGATATGGATGAAGCTCAAGTTATTAAT<br>AAGAAAGTTAAATAA
**EneoFAD-A1b**	ATGTCTCATCACCATCACCATCACGGATCCATGGCTCCAAATGTTACTTCTTCAGCAACAGAAGTTTTGTGTACTCCAGAAACTACATTGCATTCTTCATCTGAAATCAGAGCTGGTGTTG<br>AAAAGCCAACATTGAAGTACAGAAGAGAAATCGTTTGGCCAAACGTTGTTATGCATATATATTTGAACGTTGCTGCATTGTACGGTACTTACTTAATGTTGACATCAGCTAAAATTTTGAC<br>TGGTATTTGGGCTTTTATTTTGTACGAAGGTGGTATTTTAGGTATTACTGCTGGTGCACATAGATTGTGGTCTCATAGAGCTTACAAGGCAACATGGCAATTGAGATTGATCTTGATGTTG<br>TTGCATACTTTAGCTTTCCAAACATCAGTTCATGAATGGGTTAGAAACCATAGAGTTCATCATAAAAATTCTGATACTGATGCAGATCCACATAATGTTCATAGAGGTTTCTTTTTCACAC<br>ATTATGGTTGGATGTTGGTTAGAAAACATCCAGCTGTTAAGGAAAAGGGTAAATTGATCGATTGGTCTGATTTGGATGCAGATCAATTGTTGTTGTTCCAAAAGAGATACTACTTGATTTT<br>AATGCCAATTTTGTGTTTCTTGATTCCATCAGCTGTTCCAGTTTATTTGTGGGGTGAAACTTGGACAAATTCTTACCATATTGCTGCAGTTTTAAGACAAGTTGTTACTTTGCACATGACT<br>TTGATCACAAATTCTATCACTCATTGGCCAGATACATGGAGAAAGAGACCATACGATAAGAATATTTGTCCATCAGAAAACTTCATGGTTTCTTTATTGACTTTAGGTGAAGGTTGGCATA<br>ACTTCCATCATGTTTTCCCTTGGGATTACAAGACAGCAGAAATCGGTGGTAACAGAATTAATCCAACTACACATTTCATCGATTTCTGTGCTAGAATTGGTTGGCATACGATTTGAAAAC<br>AGTTCCAATGTCAGTTGTTAAGAAAAGAGTTGAAAGAACTGGTGACGGTACACATGAAATTTGGGGTTGGGGTGACAAGGATATGACTACATCTGAAAAGAATTTGGTTTATGTTATTAAT<br>AAGAAAGTTATGTAA

Supplementary Table 7. Primers used for site-directed mutagenesis. The table is related to fig. 4 of the main text.

Mutant	Template	Primer	Primer sequence
PsimFAD-A1a_86Ile	PsimFAD-A1a	PsiD2-86I_F	CAAGCAGGTATTTTAGGTATTACAGCTGGTG
		PsiD2-86I_R	GTAATACCTAAAATACCTGCTTGGTATAACAAAAC
PsimFAD-A1a_236Leu	PsimFAD-A1a	PsiD2-236L_F	GAACATGACTTTGTTGGTTAATTCAGCTGCAC
		PsiD2-236L_R	GAATTAACCAACAAAGTCATGTTCAATGTAAAAGTG

SUPPLEMENTARY REFERENCES

Bai Y, McCoy JG, Levin EJ, Sobrado P, Rajashankar KR, Fox BG, Zhou M. 2015. X-ray structure of a mammalian stearoyl-CoA desaturase. *Nature* 524:252256.

Buček A, Matoušková P, Vogel H, Šebesta P, Jahn U, Weißflog J, Svatoš A, Pichová I. 2015. Evolution of moth sex pheromone composition by a single amino acid substitution in a fatty acid desaturase. *Proc Natl Acad Sci U S A.* 112:12586–12591.

Buček A, Vazdar M, Tupec M, Svatoš A, Pichová I. 2020. Desaturase specificity is controlled by the physicochemical properties of a single amino acid residue in the substrate binding tunnel. *Comp Struct Biotechnol J.* 18:1202–1209.

Buser HR, Arn H, Guerin P, Rauscher S. 1983. Determination of double bond position in mono-unsaturated acetates by mass spectrometry of dimethyl disulfide adducts. *Anal Chem.* 55:818–822.

Cai Y, Yu XH, Chai J, Liu CJ, Shanklin J. 2020. A conserved evolutionary mechanism permits Δ9 desaturation of very-long-chain fatty acyl lipids. *J Biol Chem.* 295:11337–11345.

Ding B-J, Carraher C, Löfstedt C. 2016. Sequence variation determining stereochemistry of a Δ11 desaturase active in moth sex pheromone biosynthesis. *Insect Biochem Mol Biol.* 74:68–75.

Fay L, Richli U. 1991. Location of double bonds in polyunsaturated fatty acids by gas chromatography-mass spectrometry after 4,4-dimethyloxazoline derivatization. *J Chromatogr A.* 541:89–98.

Meesapyodsuk D, Qiu X. 2014. Structure determinants for the substrate specificity of acyl-CoA Δ9 desaturases from a marine copepod. *ACS Chem Biol.* 9:922–934.

Vanhercke T, Shrestha P, Green AG, Singh SP. 2011. Mechanistic and structural insights into the regioselectivity of an acyl-CoA fatty acid desaturase via directed molecular evolution. *J Biol Chem.* 286:12860–12869.
